# “Where do I even start?” Recommendations for faculty diversifying syllabi in ecology, evolution, and the life sciences

**DOI:** 10.1002/ece3.9719

**Published:** 2023-01-03

**Authors:** Tolulope I. N. Perrin‐Stowe, Melissa Horner, Jaime J. Coon, Lauren R. Lynch, Alida de Flamingh, Nathan B. Alexander, Elizabeth Golebie, Timothy M. Swartz, Alyssa C. Bader, Samniqueka J. Halsey

**Affiliations:** ^1^ Wisconsin Institute for Discovery Madison Wisconsin USA; ^2^ Department of Sociology University of Missouri Columbia Missouri USA; ^3^ Department of Biology Earlham College Richmond Indiana USA; ^4^ Department of Environmental Sustainability Earlham College Richmond Indiana USA; ^5^ Department of Natural Resources and Environmental Sciences University of Illinois at Urbana‐Champaign Champaign Illinois USA; ^6^ Carl R. Woese Institute for Genomic Biology University of Illinois at Urbana‐Champaign Champaign Illinois USA; ^7^ Department of Biology, Center for Biodiversity Temple University Philadelphia Pennsylvania USA; ^8^ Department of Anthropology University of Colorado Boulder Boulder Colorado USA; ^9^ Applied Computational Ecology Lab School of Natural Resources University of Missouri Columbia Missouri USA

**Keywords:** anti‐colonial, anti‐racist, ecology, environmental sciences, evolution pedagogy, instructor resources

## Abstract

Diversifying curricula is of increasing interest in higher education, including in ecology and evolution and allied fields. Yet, many educators may not know where to start. Here we provide a framework for meeting standard curriculum goals while enacting anti‐racist and anti‐colonial syllabi that is grounded in the development of a sustainable network of educators. In addition to highlighting this professional learning process and sharing the list of resources our group has developed, we provide suggestions to help educators highlight contributions of minoritized groups, explore multiple ways of knowing, and perform critical assessments of foundational views of life and environmental science fields. We further discuss the key classroom dynamics that affect the success of such anti‐racist and anti‐colonial initiatives. The retention and success of minoritized students in ecology and evolution depends on whether we address injustices in our fields. Our hope is that our fellow educators will use this paper to catalyze their own efforts to diversify their courses.

## Graphical abstract

Diversifying curricula is of increasing interest in higher education, including in ecology and evolution and allied fields. Here we provide a framework for meeting standard curriculum goals while enacting anti‐racist and anti‐colonial updates to syllabi and we share the list of resources our group has developed. Our hope is that our fellow educators will use this paper to catalyze their own efforts to diversify their courses.

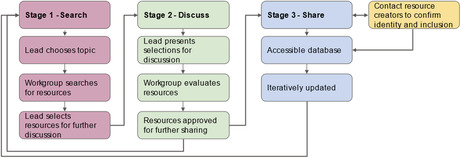

## 1 WHY DIVERSIFY CLASSROOM CONTENT?

Recently, there have been impassioned calls within a diversity of life and environmental science fields to bring anti‐racism (the process of actively identifying and opposing racism through changed policies, actions, and beliefs) into practice within our disciplines and classrooms, including in ecology and evolution (Sealey et al., 2020). Suggestions include highlighting Black women scholars, hidden figures in our fields of study (Miriti et al., 2020) and elevating Black, Indigenous, and People of Color (BIPOC) scholars through inclusion in course syllabi (Halsey et al., 2020). Yet, the need to diversify syllabi in the life and environmental sciences by elevating the voices and ideas of BIPOC is anything but new (Kimmerer, 2002, 2012; Ortega et al., 2006), and marginalization and “chronic underrepresentation” is a continuing crisis (Miriti, 2021). One reason (among many others) for insufficient progress, may be the difficulty of putting inclusive teaching theory into practice, especially for lone educators seeking to redesign classroom materials. Although many of our colleagues acknowledge the benefits (Chen & Xiao, 2021) and are eager to engage in diversifying syllabi and increase inclusion, some may be rightly worried that their solitary efforts could unintentionally exacerbate harm or be ineffective at addressing structural barriers.

The benefits of diversity to academia and research have been acknowledged (Dasgupta & Stout, 2014; Swartz et al., 2019) but many postsecondary educators' still struggle to adapt curricula and pedagogy to the increasingly diverse population of students in our classrooms (Padayachee et al., 2018). There are also educators who resist diversity, equity, inclusivity, and justice (DEIJ) initiatives in the life sciences (Iyer, 2022). Both the lack of experience and resistance hinders universities in meeting their mission to prepare students to meet the needs of an ever‐changing world (Griesel et al., 2009). Furthermore, pedagogy that does not foster inclusivity by representing the lived experiences of minoritized students, alienates these students from their academic environment and that lack of social belonging impedes their transition to and success in higher education (Criser & Knott, 2019; Padayachee et al., 2018; Patterson Silver Wolf et al., 2021). Such impacts can be long‐term and reduce the recruitment of minoritized students to the next generation of scientists and science educators (Jimenez et al., 2019; Wong‐Villacres et al., 2020).

Counteracting disparities in student success and representation within the life sciences requires addressing the cultural biases embedded within institutional practices. These practices contribute to inequities in engagement along historically marginalized demographics such as race, ethnicity, and gender (Kozlowski et al., 2022; Miriti, 2020). A starting point for this work is developing syllabi that challenge harmful paradigms within disciplines and highlight contributions from a diversity of scientists. Such efforts will encourage students and instructors in ecology, evolution, and allied fields to develop multiple historical perspectives and awareness of global issues, strengthen cultural consciousness and intercultural competence; combat prejudice and discrimination; and build social action skills that lead to change (Bennett, 2007; Molina Roldán et al., 2021; Sanger, 2020). Here, the authors (positionality shown in Box 1) propose a collaborative framework for educators to diversify their ecology and evolution syllabi. We also provide a theoretical background and a practical framework for a professional learning community and provide examples of resources, resource evaluation, and syllabi for those undertaking the vital work of anti‐racist and anti‐colonial teaching.

BOX 1 **Authors' positionality statement**

Box 1 includes the joint positionality statement of the authors of the manuscript.

Our positionality encompasses our lived identities and worldviews as scholars and includes our approach to research and teaching (Holmes, 2020). The authors are a group of early career scientists (professors, postdoctoral researchers, and doctoral students) at settler‐colonial academic institutions instructing courses at both small teaching focused colleges and research focused institutions. Authors include Black, White, and biracial (Métis/Anishinaabe and White, Tsimshian and White) identities. We are cisgender, gay, nonbinary, queer, gender nonconforming, and straight. Though our fields of study are diverse, encompassing anthropology, conservation biology, conservation psychology, disease ecology, genetics, genomics, sociology, and wildlife ecology, we are united by a shared interest in teaching and developing inclusive curricula. A combined and detailed land acknowledgment by all authors is included in File S3. In this manuscript and the related supplementary materials, we primarily discuss diversity and inclusion in relation to race, ethnicity, and settler colonial dynamics. However, these do not fully encompass the identities that should be included in diversifying work. Furthermore, many people exist at intersections of various identities which gives them unique worldviews and experiences (Crenshaw, 1989); thus, an understanding of intersectionality is needed for inclusive teaching.

## 2 WHAT IT MEANS TO DEVELOP AN ANTI‐COLONIAL SYLLABUS VS A DECOLONIAL ONE?

Creating inclusive college classrooms within the life and environmental sciences entails developing course materials that diversify content and divest from settler colonial agendas while delivering the necessary scientific curriculum. Settler colonial agendas, knowledge, and paradigms seek to reinforce settler colonialism, which is an ongoing system of power that perpetuates the genocide, repression, and erasure of Indigenous Peoples and cultures. The active effort to move away from this paradigm in academia is often referred to as “decolonizing the syllabi” but may more accurately be considered an “anti‐colonial” effort. The process of decolonization is complex and multidimensional, and its definition varies both within and across disciplines and contexts (Goeman, 2008; Padayachee et al., 2018; Smith, 1999; Tuck & Yang, 2012). From a pedagogical viewpoint, decolonization efforts are intended to decenter colonial knowledge (which erases and obfuscates Indigenous knowledge) and agitates colonizing ideologies (Allard‐Tremblay & Coburn, 2021) that shape curricular texts, assignments, and instruction (Absolon, 2019). However, some Indigenous scholars and community members within settler colonial nation states argue that using the language of “decolonization” in this context actually supports settler colonial agendas and dilutes the main decolonial effort which is centered on returning Indigenous lands to Native Nations (i.e., rematriation; Tuck & Yang, 2012). The use of the phrase “decolonizing education” to label pedagogies that might support degrees of DEIJ education, often fails to recognize the theft of the Native lands upon which all said schooling in a colonial state takes place. This is done by not specifically discussing the history of deliberate Indigenous exclusion, disenfranchisement and genocide. Furthermore, attempts of “decolonizing education” are often broad social justice projects that ironically neglect to recognize the historic and contemporary effects of settler colonization on Native communities in particular (Horner et al., 2021). We agree that this often incorrect and incomplete usage of the term has muddied the connection between decolonization to rematriation, and thus prefer instead to use the term anti‐colonial.

To avoid perpetuating the use of decolonization as a catch‐all term for social justice projects (Horner et al., 2021), we approach this pedagogical work from an “anti‐colonial” perspective, meaning we practice the ongoing, extended process of dismantling settler colonial power by identifying, critiquing, and divesting from colonial logics, policies, and practices that uniquely oppress BIPOC communities and individuals (Simmons & Dei, 2012). To develop anti‐colonial curricula means explicitly calling attention to the convergence of life and environmental sciences with issues of land, water, plants, animals, and the enduring relationships contemporary Indigenous Peoples and Native Nations hold with these more‐than‐human relatives.

## 3 RECOMMENDATIONS FOR DEVELOPING DIVERSE SYLLABI

To help fellow educators enact anti‐colonial syllabi in their classrooms, we outline a professional learning process focused on incorporating topics related to diversity, equity, inclusion, and justice. We draw on our own experience developing a “professional learning community” characterized by regular, periodic, small group discussions with other educators with similar priorities. The goal of conversations within these professional learning groups is to identify (1) relevant teaching resources that highlight BIPOC voices in science and (2) resources that provide alternative ideas and theories to settler colonial concepts inherent within our disciplines. Participants in this process can then integrate the resources identified through this process into their own syllabi. Example lesson plans illustrating the outcomes of this effort are exhibited in Box 2 and File S1. Before describing this process in detail, we outline several key steps educators should take when preparing to begin this work.

BOX 2 **Example genetics and conservation biology lessons**

**FIGURE 1:**
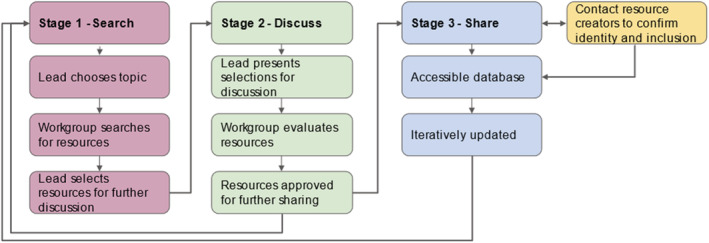
Stages of creating and sharing a resource list to build diverse and anti‐colonial syllabi. A process to aid instructors working toward diversifying undergraduate classrooms in ecology and evolution and related fields, centered on an iterative search‐discuss‐share model where faculty identify and evaluate articles, videos, podcasts, and other teaching resources. The process starts with individual responsibilities (pink), then small group responsibilities (green), and expands to a public resource (blue) which includes ensuring the author is comfortable with their resource being included (yellow).

Worked examples showing evaluation of resources from the fields of Genetics and Conservation Biology that support diverse, anti‐colonial approaches for syllabi in the life and environmental sciences, following the search‐discuss‐share process shown in Figure 1.
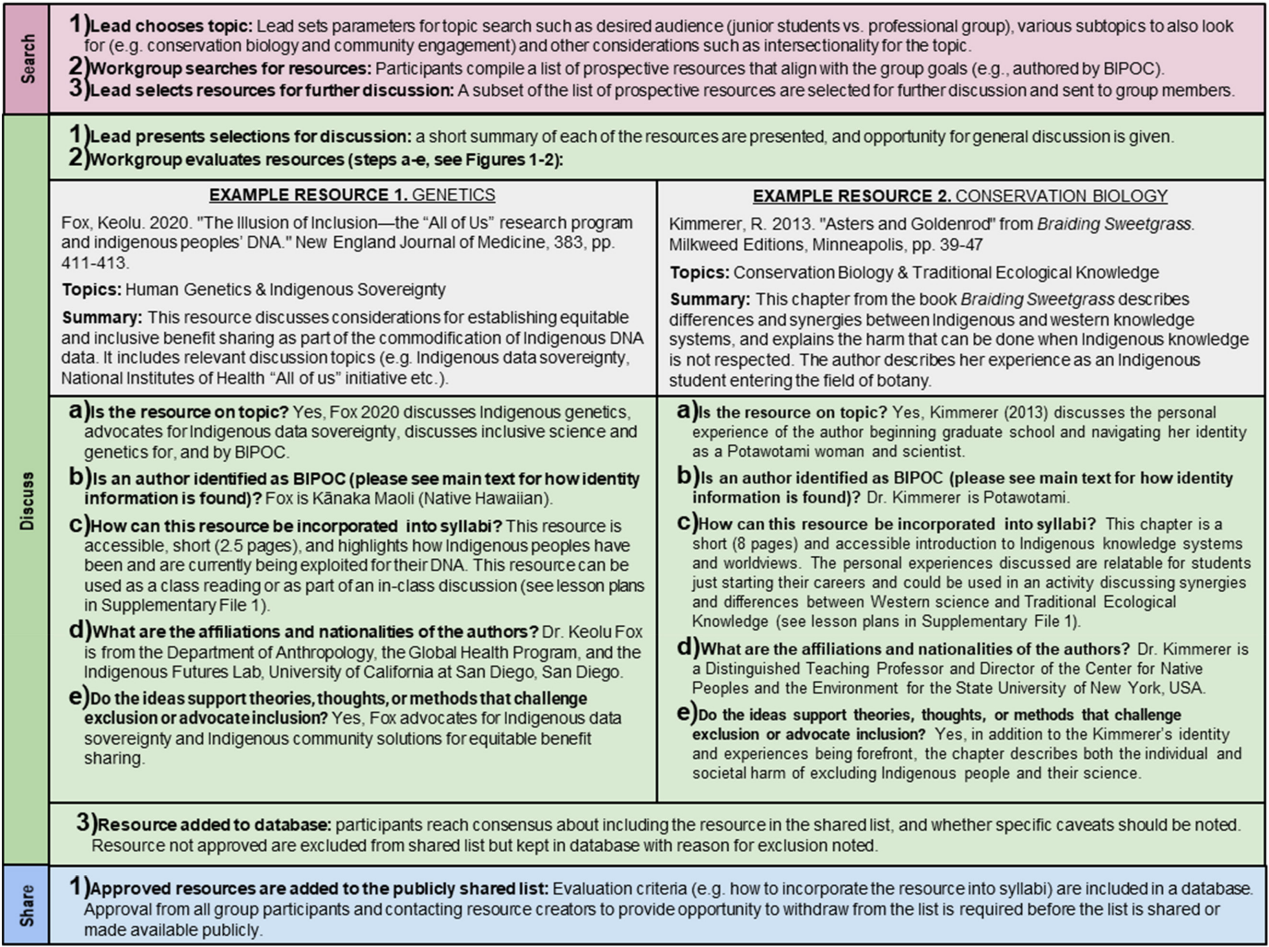

## 4 HOW DO I PREPARE?

Conversations about diversity, racism, anti‐coloniality, and social justice require preparation. The first step toward classroom transformation is to recenter your own mindset and identity as an instructor on anti‐colonial science education, and espouse empathy to students who may struggle with the same questions and challenges (Phillips et al., 2019). It is important to take the time to educate yourself about diversity, racism, settler/extractive colonization, and social justice. Avoid using lack of knowledge as an excuse to neglect addressing these topics in your class. There may be discomfort due to not fully understanding the previously mentioned concepts, as well as those of race and racism (Fuentes et al., 2019), but these conversations are crucial (Oluo, 2018). Similarly, if you have grown up in a settler colonial state (where settlers displaced the Indigenous population), you likely received an incomplete and misleading education about colonization, so you may feel unprepared to teach about anti‐coloniality (Stanley, 1998). Nevertheless, these concepts are important, and need to be discussed. We recommend educating yourself about essential concepts (such as systematic racism and implicit bias) related to DEIJ (see Table 1 for further definitions). This also includes the history of racism in your specific locale, and how racism and settler colonial ideologies have and continue to impact the dominating paradigms, the history that is shared vs. the history that is marginalized, and inclusion of past and current BIPOC in your field of study (Phillips et al., 2019). Important facets of preparation are:

1. *Think about and acknowledge your positionality*: Take time to think about your positionality (Box 1; Lichty & Palamaro‐Munsell, 2017). Think about how factors such as your race, gender, settler status (being Indigenous or a settler to a place), geographic location, sexuality, class background, and paradigms under which you were educated, impact your various privileges and how that relates to the course material. While revealing your identities with your students is a personal choice, consider discussing your positionality with your students at the beginning of the semester, especially if you share many of these identities with your students (Harbin et al., 2019). Think about how your identity has impacted your experiences, perspectives, and opportunities in life (Phillips et al., 2019; Saad, 2020). Provide examples of your connections to course material including why you think it is important to discuss issues related to diversity, racism, anti‐coloniality, and social justice. This openness may increase students' willingness to discuss these topics with you and model how to consider their own positionality as well (Cronin et al., 2021).
2. *Request expert advice*: If you have the opportunity, you should seek advice from experts in anti‐racist and anti‐colonial education. There will likely be experts at your institution who may have a wealth of resources already available. For example, you can direct questions toward to your university's office of diversity or your department's DEIJ committee, educational and/or outreach programs centered on DEIJ, and other knowledgeable staff members who have agreed to be available for this purpose. DEIJ committees and workshops at professional societies or experts in DEIJ from other institutions that can be invited for workshops, trainings, or consultations can aid in providing access to experts. We stress that it is important to appropriately compensate those that you seek expert advice from as BIPOC faculty disproportionately engage in service work surrounding DEIJ initiatives compared to their White counterparts (Rodríguez et al., 2015).
3. *Be prepared to handle (micro)aggressions*: Before teaching content related to diversity, racism, anti‐coloniality, or social justice, consider how you will handle microaggressions (subtle acts of discrimination), as well as more overt acts of discrimination that might occur during class discussion. Be prepared to interrupt and appropriately handle covert prejudice, threats, bullying, or dehumanization that occurs (Harbin et al., 2019), whether intentional or not. Some resources, cited here, have suggestions for mitigating these harmful situations which include acknowledging them when they happen and having further discussions about language, intention, and impact (Harrison & Tanner, 2018; Williams, 2020; Yoon, 2020). If you are uncomfortable having these discussions, we recommend practicing with colleagues before implementing this within the classroom.

**TABLE 1:** Key definitions

Term	Definition	References
Anti‐colonialism	A long‐term process involving economic, cultural, emotional, linguistic, academic, and psychological divesting of settler colonial power by recognizing, critiquing, and dismantling structural ideologies, practices, and policies that oppress Native Peoples	Adapted from Calliste and Dei (2000)
Anti‐racist	An action‐oriented, educational and/or political strategy for systemic and political change that addresses issues of racism and interlocking systems of social oppression	Adapted from Calliste and Dei (2000)
BIPOC	An umbrella term for Black, Indigenous, and People of Color. Note, this term is not meant to turn these identities into a monolith but is meant to acknowledge the historical and modern experiences of oppressions that these communities experience	Adapted from Halsey et al. (2020)
Decolonization	The practice of giving back lands to Indigenous communities and tribal nations (see ‘rematriation)	Adapted from Tuck and Yang (2012)
Extractive Colonialism	A form of colonization whereby “resources” (minerals, plants, humans) are taken by a colonizing nation state for the economic benefit of the colonizing nation state without setting up a permanent colonial society	Adapted from Tuck and Yang (2012)
Implicit bias	Unconscious bias linked to discrimination through automatic categorizing people according to stereotypes	Adapted from Greenwald and Banaji (1995) and Lowery et al. (2001)
Indigenous and Native Peoples	We use these terms interchangeably and use specific tribal affiliations where possible. We pluralize “Peoples” to indicate the diversity of Indigenous nations, groups, and cultures and capitalize the “P” to note that these Peoples have been practicing self‐determination and governance long before colonization	Adapted from Horner et al. (2021)
Microaggressions	Prejudices that may appear in interpersonal situations that may be microassaults (intended to hurt the intended victim through purposefully discriminatory actions), a microinsult (insensitive communication that demeans an identity), or microinvalidation (negating or ignoring the thoughts, feelings, or experiential reality of a minorized individual)	Adapted from Sue et al. (2007)
Minoritized individuals	In contrast to “minorities,” this term reflects the ongoing social experience of marginalization even when groups subject to discrimination achieve numerical majority	Adapted from Chase et al. (2014)
Rematriation	Refers to reclaiming ancestral homelands, languages, knowledges, cultures, remains, and artifacts. Used instead of the patriarchal term “repatriation.”	Adapted from Tuck (2011)
Settler colonialism	Beginning 500+ years ago, settler colonialism rapidly morphed from an event into an ongoing social, cultural, economic structure intended to control lands and construct Anglo‐European‐based societies in place of Indigenous societies. Primary aims are to erase Indigenous Peoples' cultures, governments, languages, and knowledge from the land and, to impose colonial notions of property ownership	Adapted from McKinnon (2010), McKay et al. (2020), Wolfe (2006)
Systemic racism	Refers to the well documented fact that most of our institutions—in politics, law, education, and health care, to name a few—are fundamentally organized according to assumptions and policies that work to the disadvantage of BIPOC	Adapted from Winfield et al. (2020)

*Note*: This table includes the definitions and references of select terms used in the manuscript.

## 5 A FRAMEWORK FOR COLLABORATIVE PROFESSIONAL LEARNING COMMUNITIES FOCUSED ON DIVERSIFYING SYLLABI

The above recommendations set the groundwork for moving from theory to practice in embracing anti‐racist and anti‐colonial approaches while diversifying syllabi. The process requires work on multiple scales, from individual to institutional to systemic. While working toward embodying an anti‐racist, anti‐colonial mindset on an individual level is critical, the task of creating a new syllabus that responds to diverse scholarship and students can be isolating and slow‐going. Most importantly, on our own, the biases we hold are less likely to be challenged or dismantled. Instead, developing collaborative networks, like professional learning communities, can increase capacity and reduce isolation for instructors while also increasing the longevity of important diversity initiatives (Allen‐Ramdial & Campbell, 2014; Bowne et al., 2011; Riley et al., 2019). Professional learning communities allow for the exchanging of ideas between teaching faculty and ensures that missteps in the early stages of anti‐racist and anti‐colonial work occurs alongside other instructors, and not in front of students before instructors have the tools to navigate those missteps in productive ways while teaching.

We developed a structured, instructor‐led process that facilitates collaborative work on designing diverse, anti‐colonial syllabi. The process has three goals: (1) develop a list of college‐level resources (articles, podcasts, videos, etc.) useable as teaching tools in undergraduate classrooms to help integrate topics of racial justice, traditional ecological knowledge/Indigenous knowledge systems, and environmental racism into existing life and environmental science classes; (2) seek to recognize both historical and current contributions of BIPOC to science through increased representation in syllabi, and (3) provide pedagogical resources and methodologies that support instructors who seek to include these topics in their classrooms. Although our disciplinary focus is the ecology, evolution, and environmental science, this process is extendable to other disciplines.

**FIGURE 2:**
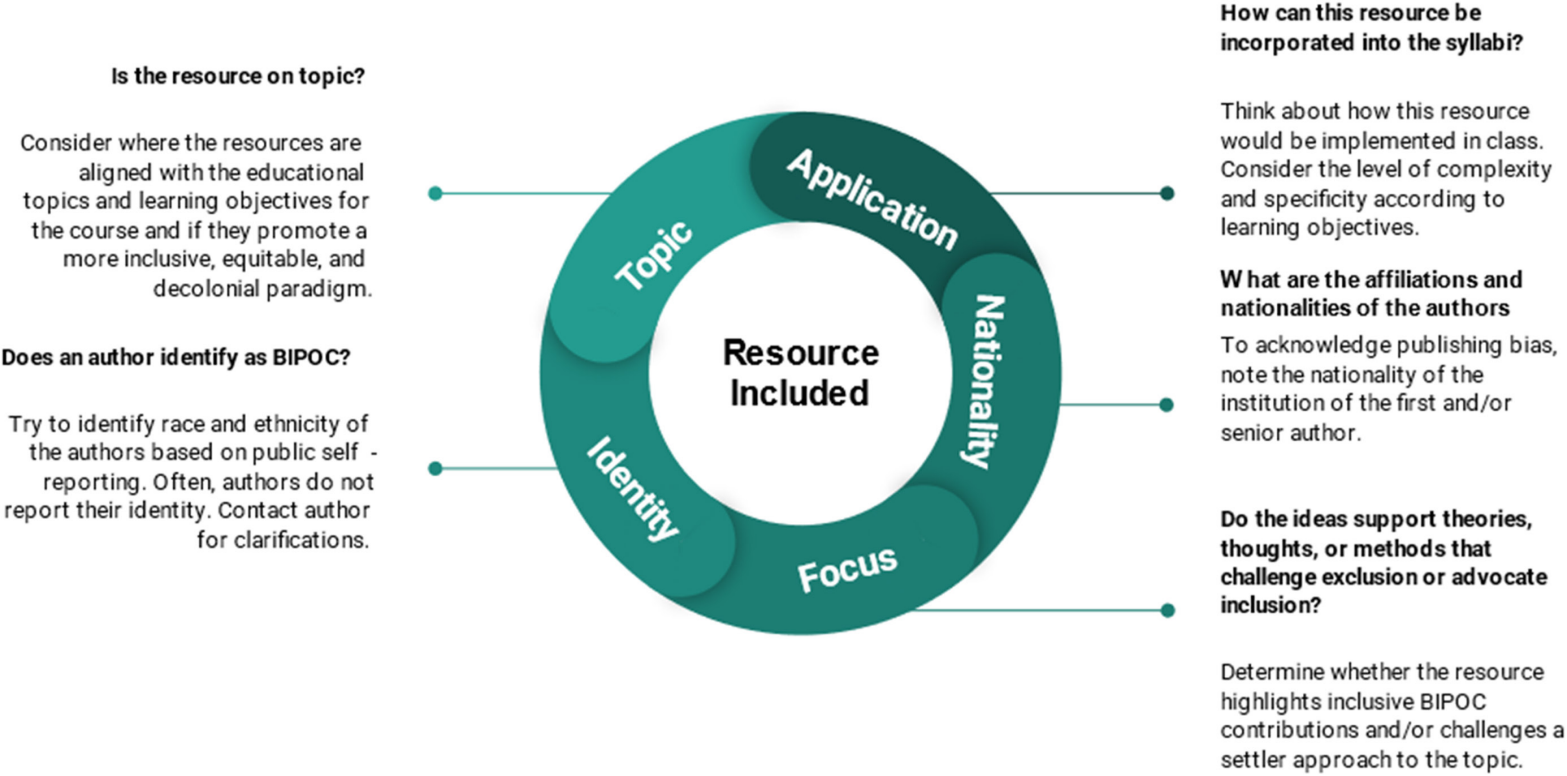
Resources to include in weekly meetings. Criteria used when evaluating peer‐reviewed articles, popular articles, videos, podcasts, and other teaching resources that work toward diversifying and anti‐colonial syllabi in ecology, evolution, and related fields.

These goals are accomplished through three stages: search, discuss, and share (Figure 1). The process is facilitated by members with relevant teaching or research experience with the leadership role alternating each session. In the search phase, each group member individually seeks resources that either (1) diversifies classroom content through centering BIPOC scientists or/and (2) works toward dismantling colonialist foundations. Resources are evaluated based on whether they are (1) on‐topic, (2) applicable to our classrooms and curriculum, and (3) are either written/created by BIPOC, originate from underrepresented identities, and/or challenge the problematic ways we traditionally teach in a particular discipline (Figure 2).

### 5.1 Stage one: Search

#### 5.1.1 Choosing course content

Once you are prepared to begin incorporating anti‐racist and anti‐colonial ideas and topics into your teaching practice, you may be wondering what content is appropriate. As you develop your syllabus, we suggest you consider the following:

1. *Highlight BIPOC inclusion, not just exclusion*: Emphasis on discrimination can be distressing to BIPOC students who may already be aware and have experience of such issues. Likewise, research about or on BIPOC but not created by BIPOC continues to center dominant and often Eurocentric perspectives (e.g., ignoring established Indigenous classification and knowledge during taxonomic assignment (Wilder et al., 2016)). Without careful consideration of these sources, prioritizing White learning over BIPOC mental health and well‐being is a risk. In other words, lessons should be planned not with the sole aim of educating students about discrimination, but to portray multiple voices in the field, especially those who have traditionally been silenced, and to indicate to BIPOC students that despite the challenges and history, they are welcome and needed in the field (Halsey et al., 2020). This ensures that BIPOC are recognized as scientists and integral contributors of the field. For example, one of the most obvious entry points to incorporating diversity in ecologically focused classes is environmental justice, which while an important topic that should not be overlooked, should also not be the only effort at diversifying your syllabus
2. *Teach the importance of multiple ways of knowing in research and education*: Many core courses often convey a mainstream narrative about the history of the field and key contributors. This approach tends to center western worldviews with a history that purposefully excludes BIPOC perspectives. The concept of multiple ways of knowing (exploration of pedagogy different to that which upholds colonial power and knowledge; Warren et al., 2020) is best introduced at the beginning of the semester and then incorporated throughout, so students are positioned to understand the value of engaging with diverse knowledge systems (Barnhardt & Kawagley, 2008; Kimmerer, 2002, 2012; Reid et al., 2021). You can find some examples of resources that you might use to introduce the concept of traditional ecological knowledge and Indigenous knowledge systems (Box 2, Files S1 and S2).
3. *Incorporate BIPOC historical figures in the field*: Carefully consider the scientists and historical figures you feature throughout the semester. The historical figures routinely taught as key and sometimes the only contributors to the development of the field tend to be White, cisgender men. Often the contributions of BIPOC, women, and people of diverse genders and sexualities are overshadowed or completely omitted (Graves, 2019; Menon, 2021). When possible, instructors should consider changing the structure of such lessons to address this long held practice and explore the work of minoritized contributors to the field. Place emphasis on sharing the contributions of BIPOC scientists and conservationists with intersectional identities (e.g., women and LGBTQ+ people).
4. *Provide a critical assessment of foundational views*: Many important and key philosophical contributions (especially in ecology and evolution) were presented by historical figures through racist and colonialist perspectives. Instructors should be careful not to present these figures and their contributions uncritically. In some cases, it may be helpful to provide background on these figures to give students context for understanding their philosophies as they are presented in other classes. For example, although John James Audubon and James Watson (among other notable figures) helped lay the foundations of their fields (ornithology and molecular biology, respectively), they also espoused or supported racist views throughout their careers (Harmon, 2019; Lanham, 2021; Nobles, 2020). Pointing out these truths can help students to separately assess the scientific achievements and merit from the harmful and often scientifically unfounded personal viewpoints perpetuated by notable scientists. It may not be necessary to spend large portions of the class covering the problematic views of figures. Still, these aspects should be considered when determining how to highlight the work of historical figures.

### 5.2 Stage two: Discuss

The most relevant resources (Figure 2) are selected by the current leader to read and discuss in detail. This next “discuss” phase is critical where problematic (e.g., racist text, supporting erasure of Indigenous Peoples, and knowledge) themes, language, or ideas are identified, and potential classroom implementations are generated (Box 2). Discussion creates a shared space to critically evaluate and reflect on one's understanding of issues, and explore new ways to teach on the given topic (Riley et al., 2019). This stage concludes with a consensus vote to enter the final stage, “share.”

### 5.3 Stage three: Share

The share stage ensures that this process has impacts beyond one or two classrooms. We recommend that the very best resources be organized and collated in a way that can be easily shared via online repositories (i.e., Google Drive, OneDrive, Dropbox) with other educators (e.g., File S2). Note that this entire process is iterative, with new resources being continuously found, critically evaluated, discussed, and shared over time. We provide two examples that show the process of how the professional learning group could evaluate a resource that is being considered in undergraduate classrooms (there is a genetics and conservation biology example; Box 2). One of the more challenging aspects of this process is finding resources (due to the disparities in numbers of BIPOC researchers and publications that are the focus of this DEIJ work) that meet these evaluation criteria.

In addition to exploring the networks of group members, we recommend educators look for publicly available lists of BIPOC in the relevant disciplines, such as the Indigenous Science Statement from the March for Science (https://www.esf.edu/indigenous‐science‐letter/). If there are no public lists relevant to the topic, contact professional organizations in the discipline and ask if there are relevant listservs (e.g., the Ecological Society of America's Black Ecologist Section Listserv). Public facing science Twitter accounts such as the Society for Advancement of Chicanos/Hispanics and Native Americans in Science (@SACNAS) or Twitter initiatives like #BlackinSTEM, #CiteASista, #CiteBlackWomen, #NativeInSTEM, and #BlackBirdersWeek are other useful ways to identify contributions from BIPOC in a given area of study (Jules & Scherrer, 2021). Members of such organizations or social media users engaging with these hashtags are likely to provide insight as well as share access to your list of resources and those created by the communities whose viewpoints you are seeking to include in your syllabus.

The benefits of our proposed learning community model and search‐discuss‐share approach are numerous. First, it reduces the burden on individual faculty, who may find the process of collating and screening such resources on their own overwhelming. It also creates space for discussion that mitigates the influence of individual bias, allowing faculty to move forward on their own anti‐colonial and anti‐racist journeys (Burke & Collier, 2017). The sharing phase allows for impact beyond individual classrooms, departments, and universities (Halsey et al., 2020). Several important factors (such as time commitments, duration of meetings, and accessibility of resources) must be considered to ensure that this model is productive, helpful, and ultimately sustainable. It is important to use a model that can be maintained, reevaluated, and updated so efforts do not fade overtime. To increase the long‐term sustainability of our process, we decided to meet bi‐weekly to discuss resources, with the off week to find resources. We reevaluate our goals, schedule, and priorities every semester so that group members are able to continue realistically participating. To further increase sustainability of such endeavors, groups should avoid harmful overreliance on BIPOC labor (referred to as the minority tax (Rodríguez et al., 2015; Trejo, 2020)) and should provide adequate compensation when BIPOC are consulted, through monetary means, letters of support for tenure evaluations, and/or through reduction in service or teaching loads (Jimenez et al., 2019).

## 6 CONSIDERATIONS DURING IMPLEMENTATION

### 6.1 Navigating the identities of the people behind the resources

Faculty engaging in this process should be aware of the potentially fraught issues associated with finding a resource creator's identities. Identity constructions are complicated, context dependent, and can shift over time (Stokes‐Brown, 2012; Weaver, 2015). People are experts regarding their own identities and experiences, and it is important for individuals with relative privilege to defer to this expertise (Wagaman, 2016). For this reason, we recommend relying upon identities as described by authors in interviews, biographies on their personal or lab websites, or by directly contacting the resource creator. Crucially, names, physical appearance, or any information not directly from the resource creator should not be used to determine identities when searching for resources to add to your syllabi (Canessa, 2007; Mislove et al., 2021). We recommend confirming author identities and allowing them to opt out of having their resource included in a shared list (Figure 1‐ yellow; see Files [Link], [Link]).

### 6.2 Engaging your institution

The ongoing actions of creating diverse and anti‐colonial syllabi can be an informative, challenging, and rewarding process. However, some educators can expect to encounter pushback from their institutions. When engaging in DEIJ efforts, it is important that conversations are represented across a hierarchy of institutional levels to ensure progress (Aguilar & Johnson, 2017). Here, we divide expectations into four categories using a modified framework representing power structures (Knutson et al., 2022). We begin by discussing relationships that have limited power dynamics (thus are less likely to result in negative consequences while navigating learning about difficult material), such as self‐education, and professional learning communities. Following this, we discuss settings where instructors both have and lack power, focusing on diversifying strategies that can be implemented in the classroom, and with administration.

### 6.3 Educating yourself

Self‐education requires instructors to assess their limitations toward implementing DEIJ principles in the classroom. BIPOC students perform better and are more motivated in courses taught by professors who believe academic ability results from successful evidence‐based pedagogical strategies and quality mentorship (Canning et al., 2019). Evaluating one's perceptions of teaching, intelligence, and one's willingness to learn and employ different pedagogical strategies should be a part of the self‐reflection when engaging with DEIJ initiatives. We find challenging one's assumptions and acknowledging biases is required to create an anti‐racist and anti‐colonial syllabus and be an effective educator. Learning and challenging dominant paradigms begins with reviewing relevant literature to understand the manifestation of systemic oppression in both societies at large and current fields of study. This will require leaning on the expertise of scholars in fields not traditionally associated with ecology and evolution.

### 6.4 Creating a professional learning community

Successfully building a diversified syllabus and equitable classroom involves developing a welcoming professional learning community to discuss topics related to DEIJ while being willing to engage in the unlearning of harmful paradigms. Although there is currently a paucity of research on DEIJ inclusion for faculty and staff (e.g., Aguilar & Johnson, 2017), faculty‐learning‐communities following the model we outline are shown to be effective for pedagogical and curricula change in STEM (Tinnell et al., 2019). The ideal professional learning community is an environment that allows for learning and growth, with grace for mistakes without the fear of career damage or social backlash. Professional learning groups will provide plenty of opportunities to cultivate microresistance (actions that respond to microaggressions and affirm the people and groups that they target) against microaggressions and stereotypical assumptions or statements directed toward colleagues or students (Irey, 2013). A professional learning community space may also alleviate individual harm and lessen the likelihood of continuing instances of prejudice and discrimination (Cheung et al., 2021; Torres et al., 2019). Building a curriculum with colleagues with diverse perspectives and reaching out to experts creates more holistic and equitable content for syllabi. Consider engaging with people who (willingly and openly) have an interest and experience in DEIJ work and initiatives and advocate for the proper compensation for this work. Although minoritized faculty are often expected to do unpaid work on DEIJ efforts (Aguilar & Johnson, 2017), professional learning groups can help spread that workload across multiple people to create supportive, sustainable networks.

### 6.5 Practicing mutual respect with your students

Awareness of the perceived consequences of interactions between instructors and students is necessary to effectively engage in DEIJ efforts in the classroom. Students can be reluctant to challenge the instructor or provide alternative viewpoints because of the potential risks to their academic record (Bolkan & Goodboy, 2013). Therefore, the instructors must create an atmosphere of openness by enthusiastically supporting the process of being uncertain and unpracticed in discussing sensitive topics while remaining respectful of the topic and colleagues. It is important to set ground rules of mutual respect for all aspects of interaction. Students may display strong resistance to efforts to create a diverse and anti‐colonial syllabi (Nemetz & Christensen, 1996). Be ready with thoughtful responses, statistics, and relevant examples to demonstrate the historical and current impacts of systematic oppression and exclusion (Gosztyla et al., 2021). This will help guide students in interrogating the paradigms that lead to their perceptions. When discussing DEIJ topics, acknowledge to students that discomfort and lack of willingness to engage with course material that challenges one's worldview or paradigm is common. However, if you center DEIJ issues as largely systemic, subtle, and unconscious instead of overt and individual, students may be able to focus on the content instead of their own discomfort. However, personal responsibility for actions and behaviors that uphold systematic oppression and the discomfort of confronting that reality should not be erased or ignored but acknowledged in a way that does not replicate harmful systems (Applebaum, 2017). Additionally, it is essential to not place the burden of explaining the harms and impacts of systemic oppression on minoritized individuals (i.e., do not ask the only BIPOC student to explain an anti‐racist or anti‐colonialist concept). The onus is on the instructor to include examples, past and present, on the impacts of exclusion of BIPOC while also informing students of the largely unacknowledged and purposely excluded history of BIPOC across academic fields of study.

### 6.6 Working with your administration

The culture or social climate surrounding current DEIJ efforts at a particular institution can be a good indicator of the genuine efforts to meaningfully diversify syllabi. Institutions that currently have DEIJ departments/initiatives may be supportive of these endeavors and have available resources to learn how to recreate syllabi with DEIJ as a central component to the course content. Institutions focused on increasing equity should be engaged in practices such as developing rubrics to assess diversity statements early in the faculty hire process (Bhalla, 2019). However, some universities will not have DEIJ initiatives in place and may even display resistance to including anti‐racist and anti‐colonial course content. Providing peer‐reviewed literature that advocates for incorporating DEIJ in the curriculum with a focus on anti‐colonialism and the diversity of lived experiences and viewpoints can encourage administrators to support initiatives. Administrations should also be made aware of policies or metrics regarding faculty, staff, and students that are outdated and prone to replicating systemic injustices. As an example, discussions with administrators should occur regarding student evaluations which are repeatedly shown to have bias against non‐male and non‐White educators (Chávez & Mitchell, 2020; Kreitzer & Sweet‐Cushman, 2021), especially regarding courses that incorporate topics related to race and gender, which results in unjust penalization for promotion and tenure evaluations (Kreitzer & Sweet‐Cushman, 2021). Recent efforts recommend more holistic efforts by administrators to incorporate diversity, mentorship, and other metrics often ignored by traditional STEM considerations of a researcher's impact (e.g., Davies et al., 2021) and adequately compensating BIPOC individuals doing DEIJ work (Jimenez et al., 2019). A primary consideration for institutions should be to support the hiring of DEIJ consultants whose scholarship embodies this work, instructors can support and recommend this practice while on hiring or diversity committees.

## 7 PUTTING EVERYTHING IN PRACTICE

As you (re)design your course, we recommend the following:

1. *Start your courses with a land acknowledgment*: Acknowledging Indigenous Peoples past, present, and future will call attention to the role that many institutions of higher education play in settler colonialism. Indeed, expropriated Indigenous lands are the foundation of the United States' land‐grant university system (Ahtone & Lee, 2021). A land acknowledgment catalyzes critical thinking regarding anti‐colonial approaches to the intersection of land and higher education (an example of a land acknowledgement can be found in File S3). Native Peoples should be referred to in the present tense, while recognizing the differences between western and Indigenous relationships to land. The impact of the statement on any Indigenous students (statements that may make them feel isolated or singled out should be avoided) in your class should be considered (Native Goverence Center, 2019). Keep in mind that reading a land acknowledgment should be viewed as a starting point that should be followed up with recommendations for engaging with local Indigenous researchers, professional societies, and class activities that encourage your students to thoughtfully consider the concept of settler colonialism and its impacts (Native Goverence Center, 2019; Wark, 2021). To ensure that this is not limited to a lip‐service statement (e.g., Sidorova, 2020), include discussions and activities that teach students to listen, both to the land through time spent in nature and to Indigenous voices through guest lectures, videos or other Indigenous‐created media (e.g., the Akiikaa project at the University of Toronto (Mashford‐Pringle & Stewart, 2019); note this knowledge will change depending on locale). Students should be provided with opportunities to develop relationships with the land, with emphasis on the validity of multiple ways of knowing, and learning about the interactions between power, privilege, and relationships with land (Blenkinsop & Fettes, 2020). Though outside the scope of this paper, there are a myriad of calls for land acknowledgement statements to be recognized as a mere entry point to broader, action‐oriented commitments to Indigenous Peoples, lands, and sovereignties (Hughes, 2019; Stewart‐Ambo & Yang, 2021). Lastly, separate statements that recognize that an institution (where applicable) has profited from and been involved with slavery (Rainville, 2019) and the continued impact on communities displaced due to institutional expansion (Chen et al., 2021) should also be considered and discussed for the purpose of conveying how connected the issues of racism and colonialism are to the very institutions where students are learning and often living.
2. *Invite Guest Speakers*: Inviting guest speakers to your class is a great way to expose your students to researchers and professionals of diverse backgrounds and expertise. It can also be a way to address a topic about which you might not feel fully equipped to lead a conversation. Keep in mind that an enormous burden is often placed on BIPOC to do the work of diversifying their institutions, and their anti‐racism work and knowledge tends to be undervalued (De Welde, 2017). Thus, be sure to properly compensate guest speakers for their time and expertise.
3. *Focus on facilitation*: You do not need to be an expert on diversity, racism, anti‐colonialism, or social justice to facilitate a productive conversation, although consulting experts can help you avoid causing confusion and misinformation. Anticipating that your students will have varying degrees of knowledge of the role that anti‐racist and anti‐colonial work play in your field and having responses, additional resources, and allowing room for clarifying questions can be things to consider and prepare for in order to facilitate these discussions. Also be willing to learn from your students experience and knowledge (Phillips et al., 2019).
4. *Avoid placing burden on BIPOC students*: Do not rely on BIPOC students to contribute disproportionately to these portions of your class. Instead, think about how you can foster effective class discussion without placing the emotional or educational burden primarily on BIPOC students (Phillips et al., 2019). Pressuring students of color to share their experiences with bigotry and oppression and using those experiences as learning opportunities in the class deprioritizes the needs of students of color and can harm their learning and well‐being (Blackwell, 2010).
5. *Expect to make mistakes*: When engaging in conversations about diversity, racism, settler colonialism, or social justice, it is certain that you will make mistakes however experienced or well‐intentioned you may be (Oluo, 2018). Minimize harm by being willing to acknowledge your mistake, apologizing, and committing to doing better going forward. Explain that you will be conducting yourself in this manner to set expectations and provide a model for students to follow.
6. *Collect student feedback*: Collecting anonymous student feedback can help you to identify strengths as well as areas for improvement in your teaching of content related to diversity, racism, settler colonialism, and social justice. Be sure to read this feedback, determine if and how it can help the course (some feedback may be indicative of individual frustration and discomfort and not necessarily reflect the experience of most students) and adjust accordingly if needed. This is not a static process and requires a continuation of feedback and adjusting course content. Ask students to reflect on what they have learned; this can be done by comparing an informal assessment of course content at the beginning of the course versus the same one completed at the end. This will help highlight to students that they have gained new perspectives and understanding.

## 8 CONCLUSION

Here we have provided a framework to create more diverse and anti‐colonial syllabi by establishing a professional learning process that we have found through our own experience aids individual instructors through collective efforts. A more diverse and holistic understanding of ecology, evolution, and allied fields can be the first step in helping future professionals to address disparities and damaging practices that are still prevalent in their fields (Fox et al., 2020; Hoffman et al., 2016; Khan & Mian, 2020; Ortega & Roby, 2021), work to remove barriers for minoritized students and faculty in STEM (Cheung et al., 2021; Kiesling et al., 2020; Miriti, 2020) and provide land management plans and inclusive ecological practices and studies (Schell et al., 2020a) that support people and the environment without replicating past harms (Frainer et al., 2020; Trisos et al., 2021). Furthermore, we propose that efforts based on collaboration and communal support (Tzou et al., 2019), increasing longevity and effectiveness of these efforts.

The framework presented here fosters a community of instructors who continuously work on challenging their world views to become better educators. Here, we emphasize that simply adding some of the resources listed here to a syllabus is a good start but is not enough. Educators have a responsibility to advocate for initiatives that benefit the community of minoritized students and faculty. In anti‐racist and anti‐colonialist pedagogy, the step from reflection to organizing is critical to enact social change (Kishimoto, 2018). The impact of scientific research and education goes far beyond the institutions they originate from, and so do our actions as professionals within this field. We call for administrations and leadership to support this work, including providing appropriate compensation and acknowledgment of this work during promotion and tenure review (Bhalla, 2019; Thomas & Nguyen, 2021). Viewing this work as necessary is especially important and administrator should consider regularly overburdened BIPOC faculty who often provide critical insights based on lived experiences. As mentioned, funding should be provided and made available to compensate BIPOC from all academic levels for their work and expertise in educational or administrative DEIJ initiatives. This added framework is one additional step to previous recommendations (Bhalla, 2019; Cronin et al., 2021; Davies et al., 2021; Fulweiler et al., 2021; Halsey et al., 2020; Knutson et al., 2022; Kozlowski et al., 2022; Schell et al., 2020b) to help to foster a more inclusive scientific community.

## AUTHOR CONTRIBUTIONS

**Tolulope Perrin‐Stowe:** Conceptualization (equal); supervision (lead); writing – original draft (equal); writing – review and editing (equal). **Melissa Horner:** Conceptualization (equal); writing – original draft (equal); writing – review and editing (equal). **Jaime J. Coon:** Conceptualization (equal); writing – original draft (equal); writing – review and editing (equal). **Lauren R. Lynch:** Conceptualization (equal); writing – original draft (equal); writing – review and editing (equal). **Alida de Flamingh:** Conceptualization (equal); writing – original draft (equal); writing – review and editing (equal). **Nathan B. Alexander:** Conceptualization (equal); writing – original draft (equal); writing – review and editing (equal). **Elizabeth Golebie:** Conceptualization (equal); writing – original draft (equal); writing – review and editing (equal). **Timothy M. Swartz:** Conceptualization (equal); writing – original draft (equal); writing – review and editing (equal). **Alyssa C. Bader:** Conceptualization (equal); writing – original draft (equal); writing – review and editing (equal). **Samniqueka Halsey:** Conceptualization (equal); funding acquisition (lead); writing – original draft (equal); writing – review and editing (equal).

## ACKNOWLEDGEMENTS

See supplement 3 for a detailed combined land acknowledgement of the Indigenous Peoples who practice/d culture, ceremony, and life on this sacred and ancestral ecology of lands and waters we occupy in the geographic areas colonially known as “Missouri”, “Illinois”, “Indiana”, “Wisconsin”, “Colorado”, and “Pennsylvania.” We also thank the reviewers who took the time to thoughtfully review this manuscript.

## CONFLICT OF INTEREST

The authors declare no conflicts of interest.

## FUNDING INFORMATION

There is no funding information for this article.

## Supporting information


File S1

File S2

File S3

File S4

File S5

File S6

File S7

## Data Availability

There was no data used for statistical analysis in this publication. The resource list that is mentioned in the manuscript is linked here, tinyurl.com/DACS‐list, and is continuously updated. Additional resources are also included as supplementary files.
