# Supplementary material for: “Where do I even start?” Recommendations for faculty diversifying syllabi in ecology, evolution, and the life sciences
Source: Ecol Evol. 2023 Jan 3;13(1):e9719. doi: 10.1002/ece3.9719 (PMC9810791; doi:10.1002/ece3.9719)
Supplement: Supplementary file 1 — File S1 [file ECE3-13-e9719-s005.pdf]

Supplementary File 1: Diverse and Anti-colonial Syllabi Example Lesson Plans

INFORMATION	CONTEXT & SEMESTER COURSE GOALS
**Course:**<br>Human Genetics (200/300 level)<br><br>**Topics:** Bioethics and human genome research<br><br>**Developed by:**<br>Alyssa C. Bader & Alida de Flamingh	*These lessons include concepts, assignments, activities, and readings that could be integrated into existing curriculum and are intended to be spread over many days/weeks. These lessons may be used in class sizes from 20-70.*<br><br>1. Understand the diversity of information that is part of human genomes, and how that information is used in biomedical research and applications<br>2. Recognize and discuss how unequal representation in both large scale genomic datasets and the research process impact historically excluded peoples, and how this relates to biomedical research and applications<br>3. Evaluate and discuss bioethical considerations of human genome research

TOPIC 1: BIOETHICS AND HUMAN GENOME RESEARCH

LEARNING OUTCOMES & CONTEXT

1. Understand, explain and give examples of key ethical considerations as they relate to human genomic research.
2. Students should be able to critically evaluate genomic research studies, focusing on ethical considerations

READINGS & RESOURCES

1. Fox, Keolu. "The illusion of inclusion—the "All of Us" research program and indigenous peoples' DNA." *New England Journal of Medicine* 383.5 (2020): 411-413. **On resource list

2. Is race science making a comeback? - NPR news article: https://www.npr.org/sections/codeswitch/2019/07/10/416496218/is-race-science-making-a-comeback**On resource list

3. Garrison NA, Hudson M, Ballantyne LL, et al. 2019. Genomic research through an Indigenous lens: Understanding the expectations. Annual review of Genomics and Human Genetics 20:495-517.**On resource list

4. Malhi RS, Bader AC. 2019. Human paleogenomic research in the Americas: A look at current data and vision for more inclusive practices now and into the future. SAA Arch Record 19(3):16-20.

5. Popejoy AB, Fullerton SM. 2016. Genomics is failing on diversity. Nature 538(7624):161-164.

6. Nature. "Henrietta lacks: science must right a historical wrong." *Nature (Editorial)* 585 (2020): 7. https://www.nature.com/articles/d41586-020-02494-z?fbclid=IwAR0RnzpGINCtLB-foLht8Duou_Ft22nlUAm-0ctOOMEhrZQfYZvrLv6MaOk

7. The Belmont Report: Office of the Secretary, Ethical Principles and Guidelines for the Protection of Human Subjects of Research, the National Commission for the Protection of Human Subjects of Biomedical and Behavioral Research. (1979). USDHS

8. Whiteford, Linda M., and Robert T. Trotter II. *Ethics for anthropological research and practice*. Waveland Press, 2008. (RICE Guide)

Activities & Concepts

- *Lecture: Understanding key ethical consideration in human genome research*
    - Introduction into basic human research ethics - Belmont Report, Institutional Review Board etc. (*see Readings and Resources*):

        A. Boundaries Between Practice and Research
        - Define IRB and associated protocols

        B. Basic Ethical Principles

        1. Respect for Persons
        2. Beneficence
        3. Justice

        C. Applications (introduce concepts here but explain each in more detail in the follow-up sections by focusing specifically on how marginalized or historically excluded communities are impacted)
        - Informed Consent
        - Assessment of Risk and Benefits
        - Selection of Subjects
    - Informed Consent
        - Discuss the historical context of racism, exclusion, and bias
            - Readings and Resources: Is race science making a comeback? - NPR article
            - Readings and Resources: Nature 2020
        - Contextualize with historical examples e.g., eugenics, indigenous exploitation (see “For further learning” section for resources)
        - For upper-level classes, an activity associated with the informed consent module can involve the students selecting a biomedical/human genomics research paper and drafting a mock “informed consent letter” to participants.
    - Community engagement and research partnerships
        - What does community engagement mean?
        - Readings and Resources: Malhi & Bader 2019
    - Research risks: discuss risks associated with each of the categories below
        - Physical risks
        - High risk/vulnerable communities - discuss how research in general and genomic research in particular is risky for these communities/groups:
            - Children
            - Incarcerated persons
            - Pregnant individuals
            - People with varying abilities
            - Economically or educationally disadvantaged person
        - Research outcomes - link this up with some of the syllabi resources

- Data sovereignty, access and sharing
  - How are scientific data shared, stored and accessed
  - Define data sovereignty
  - Why does data sovereignty matter
- Benefits - who benefits long & short term from research
  - Readings and Resources: Fox 2020
- Circumventing ethics issues:
  - Examples of IRB/protocol implementation and strategies for upholding ethics standards
  - Dynamic consent, Biocultural labels and Biocultural Notices, Nagoya Protocol

- *Activities: Evaluating human genome research using the RICE (Reflect - Investigate - Contemplate - Evaluate) Guide*
  - *Activity #1 Lab exercise: Garrison et al 2019 & Fox 2020*
    - Instructor picks case studies on human genome research and bioethical considerations (Garrison et al 2019 & Fox 2020 provide several examples that may be used, e.g., The Havasupai Tribe in Arizona, USA, The Nuu-chah-nulth in British Columbia, Canada, Data sovereignty
    - Group based assignment - the number of case studies/examples depend on class size, ~4-6 people per case study.
    - Each group critically assesses a case study using the RICE guide
      - Readings and Resources: Whiteford & Trotter 2008.
      - The RICE Guide is a problem-solving guide for determining the courses of action that could be taken by a researcher faced with an ethical quandary.
  - *Activity #2: Homework assignment (graded writing assignment)*
    - Instructor provides two case studies for students to critically assess
      - Instructor selects case studies that are relevant to front- (consent, minority/high-risk communities, lack of community-collaboration) and back-end aspects (data sovereignty and commodification).
    - Students should respectively address front-end and back-end ethical aspects as it relates to each of the case studies (specify which aspects need to be addressed for which case study)
    - Provide guidelines for writing assignment - e.g., 1.5pg on each aspect

FOR FURTHER LEARNING

1. Video - 10-minute clip of “The Gene – an intimate history” by Ken Burns on PBS): https://tinyurl.com/y8dwjb94

2. Friedmann (2019) Genetic therapies, human genetic enhancement, and ... eugenics? Gene Therapy 26:351-353.
3. Hill et al. (2019) Genome-wide analysis identifies molecular systems and 149 genetic loci associated with income. Nature Communications 10: e5741. [link]
4. Claw, Katrina G., et al. "A framework for enhancing ethical genomic research with Indigenous communities." Nature communications 9.1 (2018): 1-7. [link]
5. Malhi, Ripan S., and Alyssa Bader. "Engaging Native Americans in genomics research." American anthropologist 117.4 (2015): 743.
6. Bardill J, Bader AC, Garrison NA, Bolnick DA, Raff JA, Walker A, Malhi RS, Summer internship for INdigenous peoples in Genomics (SING) Consortium. 2018. Policy Forum: Advancing the ethics of paleogenomics: Shifting the status quo on community consultation. Science 360(6387):384-385.

INFORMATION	CONTEXT & SEMESTER COURSE GOALS
**Course:**<br>Conservation Biology (300/400 level)<br><br>**Topics:**<br>Community-based Conservation, Multiple Ways of Knowing in Conservation, & Invasive Species<br><br>**Developed by:**<br>J. Coon for Earlham College	*These lessons include concepts, assignments, activities, and readings that could be integrated into existing curriculum and are intended to be spread over many days/weeks. These lessons have been used in class sizes from 20-70.*<br><br>1. Understand key concepts of conservation biology and be able to apply them to real life conservation issues in order to make decisions.<br>2. Recognize a diversity of human values towards biodiversity, and evaluate how those values do, or should, affect conservation decisions.<br>3. Evaluate what we can do about the largest threats to biodiversity.<br>4. Develop research and science communication skills.

TOPIC 1: MULTIPLE WAYS OF KNOWING

LEARNING OUTCOMES & CONTEXT

1. Understand synergies and divergences between scientific knowledge and traditional ecological knowledge/Indigenous knowledge systems
2. Evaluate how traditional ecological knowledge contributes to conservation and how conservation could support ethical and inclusive relationships with Indigenous communities

READINGS & RESOURCES

1. Native Land map <native-land.ca/>, and Land Acknowledgement resources <native-land.ca/resources/territory-acknowledgement/>
2. Kimmerer, R. W. (2013). Asters & Goldenrod, chapter in *Braiding Sweetgrass*.

3. "The case to recognize Indigenous knowledge as science" by Albert Wiggan for TEDx Syndey <youtu.be/X5QON5l6zy8 >
4. Guidelines for Respecting Cultural Knowledge - Alaska Natives <www.uaf.edu/ankn/publications/collective-works-of-ray-b/Guidelines-for-Respecting-Cultural-Knowledge.pdf>
5. Gwich'in Renewable Resources Board, <http://www.grrb.nt.ca/traditionalknowledge.htm>
6. How to be an ally of Indigenous-led conservation <landneedsguardians.ca/how-to-be-an-ally>

Activities & Concepts

- *Activity #1: Land acknowledgement*
  - Introducing the idea of multiple ways of knowing and Indigenous Knowledge Systems/Traditional Ecological Knowledge begins by acknowledging the violent removal of Indigenous people from their lands, in many cases in the name of conservation.
  - **Homework assignment:** Have students write a brief land acknowledgement statement for their hometowns based on the native-land.ca website.
  - Questions to ask students during class (credit: @sharensworld on Twitter):
    - Whose land do you live on?
    - What do they call themselves?
    - What was done to those people?
    - Where do they live now?
    - How can you support them?
- *Important concepts (lecture)*
  - **Traditional Ecological Knowledge** and **Indigenous Knowledge Systems:** Evolving knowledge acquired by Indigenous and local peoples over hundreds or thousands of years through direct contact with the environment.
  - Indigenous knowledge integrates multiple knowledge areas (art, religion, and science).
  - Philosophical and spiritual traditions were often developed to minimize excessive negative impacts on populations or ecosystems.
  - Local knowledge example: Ask students to think about a place they know *really well* and think about how much more they know about that place versus an outsider. Now think about knowledge about a place that goes back hundreds or thousands of years. What type of knowledge could be possible? How might it be transmitted between generations? How might this knowledge support culture and conservation?
- *Activity #2: Multiple Ways of Knowing - Kimmerer 2013 Discussion*
  - Ask students to make a Venn diagram comparing traditional ecological knowledge and Western scientific knowledge, pulling from the Kimmerer reading
    - Potential avenues of comparison: time scales, methods, spatial scales, philosophies

        - Ask students to write a question about the natural world that Western science would find challenging to answer
            - Can bring up the Asters & Goldenrod from the Kimmerer reading.
        - Possible comparisons:
            - Local knowledge and traditional ecological knowledge is produced over generations, whereas scientific knowledge is on a much shorter time scale.
            - Indigenous people tend to recognize significance of unusual or unique events - what Western science would call anecdotes. Western scientists are more likely to call Indigenous knowledge anecdotal if it runs counter to Western science.
            - Western scientific tradition tends to seek generalizable "global" solutions, whereas problems are often context-specific, and we often need local knowledge to predict local phenomena
            - **Reciprocity:** a cultural norm common in Indigenous cultures, or the practice of exchanging things with others for mutual benefit. Western science typically fails to consider the responsibility scientists owe toward study organisms and the human communities near where we conduct research.
            - Role of mythology: scientists investigate by quantifying reality, whereas storytellers personify reality. Information transmitted via stories may be similar to models that simplify the world.
    - End activity with Albert Wiggan's Ted talk (10:26)
        - Make sure to note that Indigenous knowledge has been suppressed and isn't considered valid unless verified by Western science
- *Activity #3: Applying Traditional Ecological Knowledge to Conservation*
    - Since this activity uses traditional knowledge freely shared by the Gwich'in people, it is critical that this section is introduced with Guidelines for Respecting Cultural Knowledge. Students should know they are interacting with knowledge that is generously and ethically shared, and should be treated with respect. Some guidelines:
        - "Make it a practice to ensure that all cultural content has been acquired under informed consent and has been reviewed for accuracy and appropriateness by knowledgeable local people representative of the culture in question"
        - "Make every effort to utilize traditional names for people, places, items, etc., adhering to local conventions for spelling and pronunciation"
        - "Identify all primary contributors and secondary sources for a particular document, and share authorship whenever possible"
    - Discuss extractive use of Indigenous knowledge and when/whether it is or isn't appropriate to use Indigenous knowledge.
    - Next, introduce the Gwich'in people and their territories with information sourced from the Gwich'in Renewable Resources Board.

- Finally, have students explore the traditional knowledge related to individual species from the Gwich'in Renewable Resources Board. Ask them to summarize several pieces of traditional knowledge and be prepared to describe how this knowledge is relevant to the conservation of a species.

- End this series of activities by presenting ideas from the "How to be an ally of Indigenous-led conservation" pamphlet - highlighting trusting Indigenous leadership, creating space for Indigenous voices, understanding the connection between land and nationhood, and recognizing Indigenous science.

FOR FURTHER LEARNING

1. "Tending the Wild," Documentary available through PBS <https://www.pbs.org/video/tending-the-wild-eyran3/>
2. Hernandez, J., & Spencer, M. S. (2020). Weaving Indigenous Science into Ecological Sciences: Culturally Grounding Our Indigenous Scholarship. Human Biology, 92(1), 5-9.
3. Kimmerer, R. W. (2002). Weaving traditional ecological knowledge into biological education: a call to action. BioScience, 52(5), 432-438.
4. Kimmerer, R. W. (2012). Searching for synergy: integrating traditional and scientific ecological knowledge in environmental science education. Journal of Environmental Studies and Sciences, 2(4), 317-323.
5. Ramos, S.C. (2018), Considerations for culturally sensitive traditional ecological knowledge research in wildlife conservation. Wildl. Soc. Bull., 42: 358-365. doi:10.1002/wsb.881

***TOPIC 2:* COMMUNITY-BASED CONSERVATION**

LEARNING OUTCOMES

1. Understand the benefits and costs of Community-Based Conservation.
2. Apply principles of Community-Based Conservation in case studies.

READINGS & RESOURCES

1. Montgomery, R. A., Borona, K., Kasozi, H., Mudumba, T., & Ogada, M. (2020). Positioning human heritage at the center of conservation practice. Conservation Biology.
2. "How community-led conservation can save wildlife" TED video by Moreangels Mbizah <https://www.ted.com/talks/moreangels_mbizah_how_community_led_conservation_can_save_wildlife/>
3. Uses information from The Cumberland Forest Project in Kentucky and TEnnessee <https://www.nature.org/en-us/about-us/where-we-work/united-states/kentucky/stories-in-kentucky/cumberland-forest/>

ACTIVITIES & CONCEPTS

- *Lecture & discussion: Intro to Community-Based Conservation (CBC)*
    - CBC recognizes local people's interests, needs, rights, values in natural resource management
    - A response to the failures of conservation that excludes people (and is sometimes violent toward them) and top-down management
    - Moreangels Mbizah video, 'How community-led conservation can save wildlife'
    - Have students discuss 10 tenets of human heritage-centered conservation from Montgomery (2020)
        - Which tenets do students believe are the most important?
        - Any that are surprising?
        - Anything that needs further explanation?
- *Activity: Building a park using community-based conservation*
    - First present case study of the Cumberland Forest region.
        - Migratory corridor - one of North america's most important "escape routes" as plant and animal species shift ranges to the north under climate change
        - Rare freshwater mussels (i.e., Tennessee heelsplitter, rough rabbitsfoot) in Clinch River, also black mountain salamander, Cerulean warbler
    - Tell students they work for a large, international conservation organization, and are given a very large budget from a wealthy benefactor to establish a park in the Cumberland Forest region
    - Instructions: write a plan for establishing this park on a Google Slide or large piece of paper/poster board to be shared with the class.
        - What factors related to community-based conservation will you consider as you build your park?
        - How can you increase your success in conservation biodiversity using community-based conservation?
        - What issues of social equity do you think should be considered?
        - End activity after students present and respond to each others' ideas

FOR FURTHER LEARNING

1. Lichtenfeld, L.L., E.M. Naro, and E. Snowden. (2019). Community, conservation, and collaboration: A framework for success. National Geographic Society, Washington D.C., United States, and African People & Wildlife, Arusha, Tanzania.
2. Brooks, J., Waylen, K. A., & Mulder, M. B. (2013). Assessing community-based conservation projects: a systematic review and multilevel analysis of attitudinal, behavioral, ecological, and economic outcomes. Environmental Evidence, 2(1), 1-34.

TOPIC 3: **INVASIVE SPECIES**

LEARNING OUTCOMES

1. How might an individual's worldview impact the definition of invasive species?
2. How are invasive species harming biodiversity?

READINGS & RESOURCES

1. Reo, N. J., & Ogden, L. A. (2018). Anishnaabe Aki: an indigenous perspective on the global threat of invasive species. *Sustainability Science*, *13*(5), 1443-1452.
2. Davis, M. A., Chew, M. K., Hobbs, R. J., Lugo, A. E., Ewel, J. J., Vermeij, G. J., ... & Thompson, K. (2011). Don't judge species on their origins. *Nature*, 474(7350), 153-154.
3. Simberloff, D., Alexander, J., Allendorf, F., Aronson, J., Antunes, P. M., Bacher, S., ... & Blakeslee, A. (2011). Non-natives: 141 scientists object. *Nature*, 475(7354), 1.
4. Shackelford, N., Hobbs, R. J., Heller, N. E., Hallett, L. M., & Seastedt, T. R. (2013). Finding a middle-ground: the native/non-native debate. *Biological Conservation*, 158, 55-62.
5. Coon, J. J., van Riper, C. J., Morton, L. W., & Miller, J. R. (2020). What drives private landowner decisions? Exploring non-native grass management in the eastern Great Plains. *Journal of Environmental Management*, *276*, 111355.
6. Maresh Nelson, S. B., Coon, J. J., Schacht, W. H., & Miller, J. R. (2019). Cattle select against the invasive grass tall fescue in heterogeneous pastures managed with prescribed fire. *Grass and Forage Science*, *74*(3), 486-495.

ACTIVITIES & CONCEPTS

- *Activity #1* - Define Invasive Species
  - In your group, write a succinct definition for an invasive species. What criteria should be used to classify something as invasive?
  - Are invasive species different from non-native species? Weeds? Alien species? Exotic species?
  - In discussion, bring up that definitions are human constructs that reflect the values of the definer.
- *Lecture - prevalence of invasive species worldwide, general impacts*
- *Activity #2 - Invasive species game 'How do you solve a problem like invasive species?'*
  - Setup: let's play a game! You have a new invasive species called (have the students give an adjective and a noun).
  - For each of the following situations, do we work to remove it or leave it alone? Make sure to have a justification.
    - You know nothing about the invasive species
    - You know a little about the invasive species, it hurts amphibian reproduction
    - The removal hurts amphibian reproduction more.
    - The removal is expensive.

        - You know a little about the invasive species, it hurts crop productivity.
        - The species is not actually new, it turns out it's been there for 80 years.
        - The invasive species is harmful but popular with local residents
        - The invasive species is mostly on private lands.
    - Provide some examples for these different situations, such as Cane toad in Australia (harmful but popular with some local residents)
- *Lecture - How do invasive species change ecosystems? More specific impacts*
- *Activity #3 - The invasive species debate*
    - Split class into four groups: Davis (2011), Simberloff (2011), Shackleford (2013), and Reo (2018).
    - Have students write the article's main/thesis on a Google slide. What ideas are the authors responding to/pushing back on? Include a favorite quote
- *Case Study & Discussion: invasive grasses in the central U.S.* (Maresh Nelson 2019, Coon 2020)
    - Invasive grass tall fescue (*Schedonorus arundinaceus)* reduces nest survival of grassland birds, is avoided by grazing cattle and potentially causing negative health consequences, reduces the growth and abundance of arthropods, and takes over communities of native plants.
    - Landowners are also concerned about the impacts of herbicide, most effective way to control tall fescue, and some landowners appreciate tall fescue's ability to control erosion
    - Discuss: How would Davis, Simberloff, Shackleford, and Reo approach managing invasive grasses in this context? What approaches do you think will be most effective?

FOR FURTHER LEARNING

1. Bach, Thomas Michael, and Brendon MH Larson. "Speaking about weeds: indigenous elders' metaphors for invasive species and their management." Environmental Values 26.5 (2017): 561-581.
2. Kimmerer, R. W. (2013). "In the footsteps of Nanabozho: Becoming Indigenous to place." Chapter in Braiding sweetgrass: Indigenous wisdom, scientific knowledge and the teachings of plants. Milkweed Editions.
3. Reo, N. J., Whyte, K., Ranco, D., Brandt, J., Blackmer, E., & Elliott, B. (2017). Invasive species, indigenous stewards, and vulnerability discourse. American Indian Quarterly, 41(3), 201-223.
