# Supplementary material for: “Where do I even start?” Recommendations for faculty diversifying syllabi in ecology, evolution, and the life sciences
Source: Ecol Evol. 2023 Jan 3;13(1):e9719. doi: 10.1002/ece3.9719 (PMC9810791; doi:10.1002/ece3.9719)
Supplement: Supplementary file 2 — File S2 [file ECE3-13-e9719-s006.pdf]

Developing Diverse & Anti-Colonial Syllabi - FOR SHARING

Start by reading our goals document, found at this link.

. . . .

(TAB)LE OF CONTENTS

1. CLASSROOM RESOURCES
Resources that discuss topics related to diversity or social justice in the life and environmental sciences.

2. HIDDEN FIGURES
This tab showcases BIPOC in the histories of our disciplines.

3. POTENTIALLY PROBLEMATIC FIGURES
This tab shows an alternative view of some (problematic) White figures in our disciplines' histories.

4. INSTRUCTOR RESOURCES
General resources on how to create a just and anti-racist classroom, and also resources for teaching difficult or controversial topics

5. DIVERSIFY YOUR SOCIAL MEDIA
We do so much learning and finding of resources via our social media feeds. Here is an opportunity to find some good follows!

TAB: START HERE!

GUIDE TO OUR SORTABLE CLASSROOM RESOURCES TAB	You can filter for these items!
Type	peer-reviewed article, non-peer reviewed article, video, podcast
Title	
Year Created	
Source/Creator	Includes a full APA citation for journal articles.
Length	Minutes, pages, etc.
Topic 1	A general topic that may be an undergrad course
Topic 2	A second general topic that may be an undergrad course
Suited for upper or lower level class?	Our best guess as to how this resource would fit into a lower versus upper level course. Note that many of the resources best suited for upper level courses could still be used to develop examples or content for lower level courses
Creator Identity Notes	see this document for details
Creator Identity Source Information	We have only provided information about the identity of the authors where they have publicly self-identified as having a particular identity. For transparency, this tab indicates where we found this information.
Creator's Institution's Country	To the best of our knowledge, the country of the institution in which the author worked at the time the resource was published.
1-2 sentence summary	
Suggestions for use	Our brief ideas for how these could be used in a classroom. (we are always open to suggestions!)
Link	

TAB: START HERE!

Want to fact check us? Request a change? Join our group? Otherwise contact us?

email: diversifysyllabi@gmail.com

Contributors

Jaime Coon (VAP, Earlham College)
Lauren Lynch (PhD Candidate, University of Illinois at Urbana-Champaign)
Tolulope Perrin-Stowe (PhD Candidate, University of Illinois at Urbana-Champaign)
Nathan Alexander (PhD Candidate, University of Illinois at Urbana-Champaign)
Alida de Flamingh (Postdoc, University of Illinois at Urbana-Champaign)
Samniqueka Halsey (Assistant Professor, University of Missouri)
Timothy Swartz (PhD Candidate, Temple University)
Elizabeth Golebie (PhD Candidate, University of Illinois)
Emmett Smith (Assistant Professor of Biology, Earlham College)

How have resources been vetted?
Our group tries to review, read, and discuss several resources each week, but not all resources on this list have been vetted in depth. If visitors to this compilation spot anything problematic, we encourage you to reach out to us (DiversifySyllabi@gmail.com). We also encourage thorough vetting of any resource used from this list in classes. The appropriateness of resources also depends on institutional and classroom context, including the backgrounds of your specific students.

We provided resource creators an opportunity to opt-out from this list and also asked them to correct or add information about them in the list.

What disciplines are included on this list?
We have focused on the subject areas of our group members, which includes perspectives on health, ecology, evolution, environmental science, conservation, and environmental social sciences, among other areas.

TAB: START HERE!

Type	Title	Year Created	Source/Creator	Length (page or time)	Topic 1	Topic 2	Suited for upper or lower level class?	Identity Notes *see this document for details	Identity Source Information	Creator's/ Institution's Nation	1-2 sentence summary	Suggestions for use	Link
peer-reviewed article	Anishnaabe Aki: an indigenous perspective on the global threat of invasive species	2018	Reo, N.J., Ogden, L.A. Anishnaabe Aki: an indigenous perspective on the global threat of invasive species. Sustain Sci 13, 1443–1452 (2018). https://doi.org/10.1007/s11625-018-0571-4	10	Invasive Species	Traditional Ecological Knowledge	Either	Indigenous Anishnaabe scientist	stated in listed resource	United States	Reo and Ogden (2018) discuss the perspectives of native/non-native species, and offer the Anishnaabe people's perspective on plant/animal migrations. The authors also challenge the euro-centric concepts of "invasion ecology" and mention the Anishnaabe's concern with current invasion ecology ethos and management practices.	Use to highlight an indigenous, specifically an Anishnaabe perspective when describing ecological "invasions," and offers a traditional perspective to land management of "invasive species"	https://link.springer.com/article/10.1007/s11625-018-0571-4
book chapter	In the footsteps of Nanabozho: Becoming Indigenous to place	2013	Kimmerer, R. W. (2013). Chapter in Braiding sweetgrass: Indigenous wisdom, scientific knowledge and the teachings of plants. Milkweed Editions.	11	Invasive Species	Traditional Ecological Knowledge	Either	Potawotami scientist	stated in listed resource	United States	Kimmerer discusses the idea of 'indigenous to place', talking about how people and plants have moved over time and will continue to do so. The invasive plant part of this chapter comes at the end, and White Man's Footstep (Plantago major) contrasted with garlic mustard and tamarisk.	Use to discuss the concept of nativeness and 'naturalization', and how our ideas of the role of plants may shift over time.	https://books.google.com/books?hl=en&lr=&id=vmM9BAAAQBAJ&oi=fnd&pg=PR7&dq=braiding+sweetgrass+in+the+footsteps+of+nanabozho&ots=nhmfzFa4aS&sig=IxjaF1RkcI5xbEb7V9N45veBAxs#v=onepage&q=braiding%20sweetgrass%20in%20the%20footsteps%20of%20nanabozho&f=false
peer-reviewed article	Can you hear us now? Voices from the margin: Using indigenous methdologies in geographic research	2007	Louis, R. P. (2007). Can you hear us now? Voices from the margin: Using indigenous methodologies in geographic research. Geographical research, 45(2), 130-139.	10	Landscape Ecology	Traditional Ecological Knowledge	Either	Indigenous Hawaiian scientist	stated in listed resource & personal website	United States	This article is an indigenous Hawaiian cartographer's perspective on potential ethical use of TEK in geography.	This perspective could be used to discuss how TEK could contribute ethically to landscape ecology.	https://onlinelibrary.wiley.com/doi/abs/10.1111/j.1745-5871.2007.00443.x
video	Potential of traditional fire management and carbon farming in Venezuela - Dr Bibiana Bilbao	2012	United Nations University	4:00	Landscape Ecology	Traditional Ecological Knowledge	Lower			Venezuela	In Spanish with subtitles-In this video Dr. Bilboa talks about how they have been investigating the traditional uses of fire by the Pemón people within Canaima National Park which has a savannah-forest mosaic landscape. This is relevant to the field of Lanscape Ecology because fire is used by indiginous communities as a management tool to shape ecological processes within the landscape	This might be a good video to introduce lower level classes to the use of traditional practises in managing or shaping ecological processes.	https://www.youtube.com/watch?time_continue=212&v=dXMf7T8KddE&feature=emb_logo
news article	How Decades of Racist Housing Policy Left Neighborhoods Sweltering	2020	Plumer, B., Popovich, N. NewYork Times		Landscape Ecology	Urban Ecology	Either			United States	This news article discusses redlining and the impacts on temperature. This includes graphics and visualization of drawing out the districts and then overlaying a heat map, comparing red-lined districts to predominantly white districts.	This would be a good visualization used with Schell et al. 2020 or as an introduction to how systemic racism can impact temperature in urban settings	https://www.nytimes.com/interactive/2020/08/24/climate/racism-redlining-cities-global-warming.html
Peer-reviewed article	Positioning human heritage at the center of conservation practice	2020	Montgomery, R.A., Borona, K., Kasozi, H., Mudumba, T. and Ogada, M. (2020), Positioning human heritage at the center of conservation practice. Conservation Biology.	9	Conservation Biology		Either	Coauthors are Ugandan scientist (T Mudumba) and Kenyan scientist (M Ogada)	Wildlife Conservation Network Website (https://wildnet.org/meet-wcn-scholar-tutilo-mudumba/) & author byline in The Elephant (https://www.theelephant.info/features/2020/06/12/why-black-lives-dont-matter-in-kenyas-colonial-conservancies/)	United States & Kenya	Provides a background on how conservation practices have been viewed and 10 recommendations to employ when doing community-based conservation practices focusing on heritage-based concepts.	This paper highlights 10 tenants that can be adapted for freshman level lectures. It could also be an assigned paper as it gives a good background of conservation biology methods and the need for refocusing conservation practices	https://conbio.onlinelibrary.wiley.com/doi/full/10.1111/cobi.13483
podcast	Violet Matiru, Zoologist, Researcher and Campaigner, Executive Director of MCDI Kenya	2020	Black Nature Narratives		Conservation Biology	Wildlife Ecology	Upper	Interviewee is Black Kenyan scientist	Stated in listed resource		Discusses the failure of western funding and conservation models and the in inequity they produce, and unpick the tripartite collusion of conservation, colonialism and corporations on severing people from nature whilst creating the illusion of protection and care.	Could be used in discussions of community-based conservation, problems with protected areas and Western models of conservation.	https://soundcloud.com/black-nature-narratives/s2-ep-2-violet-matiru-zoologist-researcher-and-campaigner
video	How community-led conservation can save wildlife	2019	TED video by Moreangels Mbizah		Conservation Biology	Wildlife Ecology	Either	Zimbabwean scientist	Stated in listed resource	United Kingdom & Zimbabwe	Moreangels Mbizah studied the famous Cecil the lion until he was shot by a trophy hunter in 2015. She wonders how things could've gone differently, asking: "What if the community that lived next to Cecil was involved in protecting him?" In a quick talk, Mbizah shares the state of conservation in her home of Zimbabwe -- and why she thinks that communities living with wildlife are the ones best positioned to help them.	Discussion of trophy hunting, inclusion of local communities<br>Very accessible - appropriate for lower level classes	https://www.ted.com/talks/moreangels_mbizah_how_community_led_conservation_can_save_wildlife?language=en
non peer-reviewed article	American environmentalism's racist roots have shaped global thinking about conservation	2020	Prakash Kashwan, The Conversation		Conservation Biology	Environmental justice	Both	Indian scientist	LinkedIn	United States	This is a popular media article that talks about how many conservation practises have racist roots.		https://theconversation.com/american-environmentalisms-racist-roots-have-shaped-global-thinking-about-conservation-143783
Peer-reviewed article	Assessing community-based conservation projects: A systematic review and multilevel analysis of attitudinal, behavioral, ecological, and economic outcomes	2013	Jeremy Brooks, Kerry Ann Waylen & Monique Borgerhoff Mulder		Conservation Biology	Community based conservation	Upper			United States	This source is a systematic review of different conservation projects that focus on the community and how successful they are in general and what aspects of the projects lead to success. I think it is a good source to use with those who are skeptical about taking the time and energy to include communitites in a comprehensive way in conservation initiatives.		https://environmentalevidencejournal.biomedcentral.com/articles/10.1186/2047-2382-2-2
video	Indigenous Communities Are on the Front Lines of Climate Change	2018	Talia Buford, ProPublica	6	Traditional Ecological Knowledge	Environmental Science	Lower	Black journalist	LinkedIn (National Association of Black Journalists)	United States	This video is the one that I use to introduce TEK as a topic to introductory courses. I think it provides a really nice nuanced introduction to the topic and so succinctly as well, which is always a bonus.		https://www.youtube.com/watch?v=xlGnve1cjOY&t=312s
video	The case to recognise Indigenous knowledge as science	2019	Albert Wiggan, TEDxSydney		Traditional Ecological Knowledge		either	Bardi-Kija-Nyul Nyul speaker	Stated in listed resource	Australia			https://www.youtube.com/watch?v=X5QON5l6zy8
video	Indigenous knowledge meets science to solve climate change	2020	Hindou Oumarou Ibrahim, TED	13:00	Traditional Ecological Knowledge	Climate Change	lower	Indigenous Mbororo speaker	Twitter Bio/World Economic Forum Bio	Chad	Very accessible and engaging intro to Indigenous knowledge, and includes an example of the speaker's work in her own community (she is a geographer and does participatory mapping, but uses very accessible language to explain)		https://www.youtube.com/watch?v=z3d_UsYgt1c
video	"Tending the Wild"	2020	KCET Link media group and Autry museum of the American West		Traditional Ecological Knowledge	Resource management	either	Several Indigenous interviewees and presenters; film consultant (Timara Lotah Link) is from Chumash Coastal Band	kcet.org (info about documentary)	United States	Tending the Wild shines light on the environmental knowledge of indigenous peoples across California by exploring how they have actively shaped and tended the land for millennia, in the process developing a deep understanding of plant and animal life. This documentary examines how humans are necessary to live in balance with nature and how traditional practices can inspire a new generation of Californians to tend their environment.	I like this documentary because it's presented by Indigenous peoples. The longer documentary has a great introduction into what TEK encompasses (first 6 minutes). There are also shorter clips/episodes on relevant topics (e.g. decolonizing the diet, cultural burning to preserve and rejuvinate landscapes, how native peoples are confronting environmental threats), which might be easier to incorporate into classroom activities?	https://www.kcet.org/shows/tending-the-wild/episodes/tending-the-wild
peer-reviewed article	"Searching for synergy: integrating traditional and scientific ecological knowledge in environmental science education"	2012	Kimmerer, R. W. (2012). Searching for synergy: integrating traditional and scientific ecological knowledge in environmental science education. Journal of Environmental Studies and Sciences, 2(4), 317-323.	7	Traditional Ecological Knowledge	Environmental Science	upper	Potawotami scientist	Stated in listed resource	United States	A follow up to Kimmerer (2002) in some ways earlier in this list, but more focused on environmental degradation. Discusses how TEK can inform natural resource planning and environmental policy, and so must appear in the education of future environmental scientists.	A great opening piece about TEK for an environmental science class. This paper is essentially a 'how to' for an instructor looking to integrate TEK into an environmental science class, but could also easily be a reading for an upper-level or grad class.	https://link.springer.com/article/10.1007/s13412-012-0091-y
non peer-reviewed article	"Women have disrupted research on bird song, and their findings show how diversity can improve all fields of science"	2020	K Omland, E Rose, and K Odom, The Conversation	4	Ornithology/Zoology	Intro Ecology	either			United States	This article describes how diversity in science benefits our learning, and gives the example of female scientists and bird song.	I would use to frame a general conversation on the importance of diversity in science.	https://theconversation.com/women-have-disrupted-research-on-bird-song-and-their-findings-show-how-diversity-can-improve-all-fields-of-science-142874
book chapter	"Asters and Goldenrod" from Braiding Sweetgrass	2013	Kimmerer, R. 2013. "Asters and Goldenrod" from Braiding Sweetgrass. Milkweed Editions, Mineapolis, pp. 39-47	8	Traditional Ecological Knowledge		either	Potawotami scientist	Stated in listed resource	United States	I think this chapter does a nice job of both describing some of the differences between indigenous and western knowledge systems, and explaining the harm that can be done when indigenous knowledge systems are not respected in academia. The author describes her experience as an indigenous student entering the field of botany.	I think this article is useful for introducing students to indigenous knowledge systems and the concept of worldviews in general. I used it as assigned reading for an asynchronous online discussion, and I think it would also work well as preparation for a synchronous class discussion.	https://milkweed.org/book/braiding-sweetgrass
video	Take What is Given to You	2013	Robin Kimmerer, Recorded video from Bioneers Conference	21:00	Traditional Ecological Knowledge		either	Potawotami scientist	Stated in listed resource	United States	Touches on a number of topics including the differences between indigenous and western knowledge systems, the gift economy, and social and environmental harms that have been done by colonists in the US	This video identifies some of the differences between indigenous and western knowledge systems from 7:47-9:57. I showed the video from the beginning to 9:57 in order to introduce indigenous knowledge systems (Kimmerer ties together multiple topics in this talk, so it made more sense if they watched the full first 10 minutes rather than just 7:47-9:57)	https://bioneers.org/robin-kimmerer-take-given-ztvz1709/
podcast	To Manage Wildfire, California Looks To What Tribes Have Known All Along		NPR story by correspondent Lauren Sommer, interviews with No. Cal. tribal land managers	5:00	Traditional Ecological Knowledge	Resource Management	either	Interviewees are Indigenous land managers	Stated in listed resource	United States	Interviews with indigenous land managers who are reintroducing prescribed burning in California.	Highlights how the problems of fires in California partly stem from suppression of fires that had been used by indigenous peoples to manage the landscape for centuries. Provides a contemporary front page news story to think about indigenous resource management efforts. I think it could be a good jumping off point for talking about how many ecological problems we face have a colonial legacy, and potentially their solutions could come from listening and learning from people whose ancestors occupied these lands for millenia.	https://www.npr.org/2020/08/24/899422710/to-manage-wildfire-california-looks-to-what-tribes-have-known-all-along
Report	Ecohealth and Aboriginal Health: a review of	2011	Margot W Parkes & National Collaborating Centre for	12	Human Health		either			Canada	This document is a summary of ecohealth projects around the globe, and discusses, for example, connections between people, place and health. 'Aboriginal' in this review refers to First Nations, Métis and Inuit peoples	This might provide a easy introduction into the concept of ecohealth, and it is also a great resource for fiding other studies	https://www.ccnsa-nccah.ca/docs/emerging/FS-EcohealthAboriginalHealth-Parkes-EN.pdf

TAB: CLASSROOM RESOURCES - PLEASE SEE tinyurl.com/DACS-list

Feature article	How to include Indigenous researchers and their knowledge	2021	Virginia Gewin	3	Responsible/Respectful Research Methods	Traditional Ecological Knowledge	either	Article features writing from several Indigenous scientists			Interviews with early-career Indigenous scientists about how colleauges and institutions can fight marginalization and include Indigenous research equitably		https://www.nature.com/articles/d41586-021-00022-1
peer-reviewed article	Structural racism and health inequities in the USA: evidence and interventions	2017	Bailey, Z. D., N. Krieger, M. Agénor, J. Graves, N. Linos, and M. T. Bassett. 2017. America: Equity and Equality in Health 3 Structural racism and health inequities in the USA: evidence and interventions. www.thelancet.com. Volume 389. <www.thelancet.com/>.	11	Human health		upper	Jamaican scientist	Interview with News@TheU (https://news.miami.edu/stories/2021/02/researcher-works-to-eliminate-the-blind-spot-of-racism.html)	United States	Discusses on how structural racism needs to be addressed to address health concerns	good tables with clear data, provides a description of pathways between racism and health.	https://www.sciencedirect.com/science/article/pii/S014067361730569X
peer-reviewed article	The geopolitical ecology of conservation: The emergence of illegal wildlife trade as national security interest and the re-shaping of US foreign conservation assistance	2020	Massé, F., and J. D. Margulies. 2020. The geopolitical ecology of conservation: The emergence of illegal wildlife trade as national security interest and the re-shaping of US foreign conservation assistance. World Development 132	15	Environmental Social Science	Illegal Wildlife Trade	upper			United Kingdom	Describes the global/US shift of conservation to focus on the Illegal Wildlife Trade and national security. This does not engage specifically with race/colonialism, but does describe a hightened increase in focusing on wildlife crimes when it comes to conservation	This would be a good partner to a paper describing why people commit wildlife crimes. Increased policing, especially in the US, is often linked to colonization and racist systems. There are good figures for describing US timeline in increasing conservation efforts on the illegal wildlife trade.	https://www.sciencedirect.com/science/article/pii/S0305750X2030084X
non peer-reviewed article	"I work in the environmental movement. I don't care if you recycle"	2019	Mary Annaise Heglar for Vox	7	Environmental Social Science	Human Behavior/Social Movements	lower	Black author	Interview in Atmos Magazine (https://atmos.earth/mary-heglar-climate-change-intersectionality/)	United States	This piece critiques the view that individual behavior is the answer to climage change.	A great discussion piece in a class that discusses environmental behavior - to what extent should the environmental movement focus on individual behaviors? Pair with specific calls to action and pairing it with a reading about how large-scale corporations are responsible for much of the world's climate woes and the ways in which we can fight to hold them accountable.	https://www.vox.com/the-highlight/2019/5/28/18629833/climate-change-2019-green-new-deal
peer-reviewed article	"Revisiting Environmental Belief and Behavior Among Ethnic Groups in the U.S."	2019	Medina, V., DeRonda, A., Ross, N., Curtin, D., & Jia, F. (2019). Revisiting environmental belief and behavior among ethnic groups in the US. Frontiers in Psychology, 10, 629.		Environmental Social Science	Social-psychology	either	POC authors	Cannot find self ID		This piece summarizes problematic viewpoints related to environmental social science that have persisted - such as that communities of color are in 'survival mode' and thus can't spend time thinking about environmental concerns. The authors combat these ideas.	This paper would be another good one for a discussion - it would work fine for a lower level class.	https://www.frontiersin.org/articles/10.3389/fpsyg.2019.00629/full
non peer-reviewed article	"Deforestation and Climate Change Threaten the Most Beloved Wild Birds"	2020	By UC Davis News and Media Relations on October 7, 2020 in Environment		Environmental Social Science	Cultural Ecosystem Services	either	Study is about attitudes toward birds in Costa Rica	Stated in listed resource	Canada & United States	This is a press article that covers a recent scientific publication. It covers interesting topics, for example, conserving culturally important species. "This study provides a novel and generalizable pathway for assessing the effects of environmental changes on cultural services and integrating the sociocultural and ecological dimensions of biodiversity."	This is a press article but it has a link to the actual scientific paper, so it could be used in both lower and upper level courses. The scientific paper is also informative and perhaps a good alternative to the press release for graduate discussion classes.	https://www.ucdavis.edu/news/deforestation-and-climate-change-threaten-most-beloved-wild-birds/
non peer-reviewed article	"Conservation's people problem"	2016	Jeremy Hance		Environmental Social Science	Traditional Ecological Knowledge	either			United States	This article looks at the history of conservation and indigenous populations and how it has changed in the past 30 years to being hostile to indigenous peoples to starting to include and even center them in conservation efforts	To give a good overview of the relationship between traditional conservation methods and various indigenous and local communities	https://news.mongabay.com/2016/05/186480/
peer-reviewed article	"From potential to practical: conserving bees in urban public green spaces"	2019	Katherine Turo and Mary Gardiner	8	Urban Ecology	Pollinators	Upper			United States	The authors argue that conservation is difficult to achieve when the aesthetic and safety concerns of urban residents are not reconciled with the goals and habitat designs of conservation practitioners.	Highlights the deep interconnections between people and nature in urban areas and how conservation in urban areas requires holistic thinking about the social and ecological challenges. The authors argue that urban conservation is tough, and is doomed to failure if it doesn't work with the social and political dynamics present.	https://esajournals.onlinelibrary.wiley.com/doi/epdf/10.1002/fee.2015
non peer-reviewed article	"Seeing himself in the science"	2020	Julie Davidow for UW Magazine	6	Urban Ecology		either	interviewee is a Black scientist (C. Schell)	ESA's Black Ecologist Listserv, stated in listed resource	United States	Describes how a researcher's Black identity enhances his science.	This paper is a nice addition to Schell et al. (2020), which is listed earlier in this document, to show the importance of racial justice to science.	https://magazine.washington.edu/feature/ecologist-christopher-schell-sees-himself-in-the-science/
poetic work	Ode to New York: A performance piece	2014	Carolyn Finney	6:00	Urban ecology		either	Black scientist & author	Interview with Central Park Conservancy (https://www.centralparknyc.org/articles/5-questions-with-dr-carolyn-finney)	United States	Creative piece that explores the question "How is nature critical to a 21st century urban ethic?"	Perhaps introducing urban ecology, getting students thinking about the relationship between humans and nature, exploring other ways of knowing.	https://www.humansandnature.org/urban-land-ethic-carolyn-finney
Blog post	Engaging High Potential Communities in Urban Nature Conservation	2018	Jensen Montambault		Urban ecology		either			United States	Blog post that summarizes findings from a peer-reviewed article that highlighted "perspectives and opportunities for authentic engagements." Reframes "under-served" as "high-potential"		https://blog.nature.org/science/2018/04/27/engaging-high-potential-communities-in-urban-nature-conservation/
Video	Housing segregation and redlining in America: A short history	2019	NPR	6:36	Urban ecology		either	Code Switch team describes itself as a "multi-racial, multi-generational team"	Code Switch website	United States	Explains what redlining is and how it has and continues to impact the well-being of people of color in cities.	The main focus of this video is not on environmental issues (although environmental justice is mentioned). However, I think that the video could be used to explain the concept of redlining to students before discussing the environmental implications of this practice.	https://www.youtube.com/watch?v=O5FBJyqfoLM
peer-reviewed article (review paper)	Biodiversity and socioeconomics in the city: a review of the luxury effect	2018	Leong, M., R.R. Dunn, M.D. Trautwein	6	Urban ecology		mid/upper level			United States	Review article describing research that has been done on the luxury effect.	Useful for describing the trend of people from higher socioeconomic statuses having greater access to biodiversity in cities.	https://royalsocietypublishing-org.proxy2.library.illinois.edu/doi/pdf/10.1098/rsbl.2018.0082
GIS dataset	Mapping Inequality	2019	University of Richmond's Digital Scholarship Lab		Urban ecology						This dataset has very well made digitized maps of redlining in US cities, in both vector formats and georeferenced maps	Could be a great resource for GIS module to explore environmental racism and compare redlining data to current tree cover, pollution, etc. dataset	https://dsl.richmond.edu/panorama/redlining/#loc=7/39.914/-73.586&text=intro
popular article	Amid protests against racism, scientists move to strip offensive names from journals, prizes, and more	2020	Cahan, E. 2020. Amid protests against racism, scientists move to strip offensive names from journals, prizes, and more. Science		Taxonomy		either				Cahan 2020 describes a movement to remove problematic language and the names of people who held racist views from scientific language	This article could be paired with Cain 2020. Could be used in a discussion of taxonomic naming specifically, or could be used in a general conversation about the impacts of the language that we use.	https://www.sciencemag.org/news/2020/07/amid-protests-against-racism-scientists-move-strip-offensive-names-journals-prizes-and
Video Game	Growing Up Ojibwe	2020	Great Lakes Indian Fish and Wildlife Commission-game creation by Eleanore Falck		Traditional Ecological Knowledge		lower				Video game that provides interactive discussions to learn about treaties, maple sap collection, spear fishing, and wild rice collection.	This would be a good supplemental tool for learning and engaging with TEK. Further resources from the commission are available here: http://www.glifwc.org/publications/?fbclid=IwAR0mS8CAvCUHzx4vStudO0uF3SkAneO_E_h2WjjVg_OAqVq7XukoxGD2LyU	https://glifwc.itch.io/growing-up-ojibwe-the-game
report	Sustainable Tribal Economies: A Guide to Restoring Energy and Food Sovereighnty in Native America	2009	Sustainable Tribal Economies: A Guide to Restoring Energy and Food Sovereighnty in Native America, by Honor the Earth	74	Sustainability	Environmental Economics	either	Publishing organization & authors include Indigenous writers (including W. LaDuke, Polish/Anishnaabe Bear Clan)	Website (https://www.honorearth.org/about) and interview with Winona LaDuke (https://www.yesmagazine.org/issue/just-foreign-policy/2008/06/18/an-interview-with-winona-laduke)	United States	"This booklet explores food and energy issues in tribal communities, recognizes their linkages, provides examples of tribal innovation and outlines options for tribal communities to create sustainable energy and food economies for this millennium and for the generations yet to come."	Although the report is long, sections could be used to give Indigenous perspectives on sustainable economies, as well as climate change, oil, fuel poverty, food insecurity, 'false solutions' (clean coal, carbon capture and sequestration, unsustainable biofuels', as well as solutions-oriented chapters.	https://d3n8a8pro7vhmx.cloudfront.net/themes/524d95ff9670a41eab000002/attachments/original/1388422349/Sustainable_Tribal_Economies_HTE.pdf?1388422349
peer-reviewed article	Softly, softly: genetics, intelligence and the hidden racism of the new geneism	2016	David Gillborn (2016) Softly, softly: genetics, intelligence and the hidden racism of the new geneism, Journal of Education Policy, 31:4, 365-388, DOI: 10.1080/02680939.2016.1139189	25	Genetics		either			United Kingdom	Discusses the issues with connecting genetics to intelligence and race. Provides a history of using intelligence as a racist evaluator and the misapplications of genetics towards intelligence	This paper provides a great background of racism in intelligence and genetic studies and why these studies should not be done	https://www.tandfonline.com/doi/full/10.1080/02680939.2016.1139189
Podcast & article	Is 'Race Science' making a comeback?	2019	NPR Codeswitch		Genetics		either	Interviewee is Indian-Punjabi	Interview with The Hindu (https://www.thehindu.com/society/we-forget-how-hard-women-have-already-fought-for-the-rights-we-have-angela-saini/article23528768.ece)	United Kingdom	Interview with Angela Saini, author of Superior, the Return of Race Science about "how race isn't real (but you know... still is), why DNA tests are misleading, and how race science crept its way into the 21st century"	There is a podcast as well as a summary article, so for a more brief overview, just the summary could be used	https://www.npr.org/sections/codeswitch/2019/07/10/416496218/is-race-science-making-a-comeback
peer-reviewed article	Toxic waste dumping in the Global South as a form of environmental racism: Evidence from the Gulf of Guinea	2020	Ifesinachi Okafor-Yarwood & Ibukun Jacob Adewumi (2020) Toxic waste dumping in the Global South as a form of environmental racism: Evidence from the Gulf of Guinea, African Studies, 79:3, 285-304, DOI: 10.1080/00020184.2020.1827947		Sustainability	Environmental Economics	either			United Kingdom and United States	The toxic waste trade (both legal and illegal) constitutes environmental racism of the global North particularly targetting the global South. Here, the authors provide examples for the regions along the Gulf of Guinea and identifies how nations use loopholes in the Basel Convention which limits international toxic waste trade, and discusses the Bamako Convention which limits toxic-waste movement within Africa.	This would be a good paper for discussions on the toxic waste trade and discussing the global North and Western nations exporting waste to other countries. Furthermore this paper connects this to international conventions, allowing a tie in to policy making. For lower-level courses, using the examples provided by this paper would be especially beneficial.	https://www.tandfonline.com/doi/full/10.1080/00020184.2020.1827947
peer-reviewed article	Income Inequality and CO2 Emissions in Developing Countries: The Moderating Role of Financial Instability	2020	Bo Yang, Minhaj Ali, Shujahat Haider Hashmi & Mohsin Shabir		Sustainability	Environmental Economics	upper			China	Paper discusses the effects of income inequality and financial instability on CO2 emissions in the presence of fossil fuel energy, economic development, industrialization, and trade openness in 47 countries. Conclusions suggest that countries should decrease income inequality and invest in renewable energies to reduce environmental degradation	Would be useful to look at the overall conclusions to determine the impact that income inequality has on global sustainability aims. Data is aggregated from a large number of countries.	https://www.mdpi.com/2071-1050/12/17/6810/htm
Podcast	In Those Genes	2019	Janina M Jeff, PhD		Genetics	Genetic Testing	either	Black female geneticist	Stated in listed resource	United States	A hip-hop inspired podcast that uses genetics to uncover the lost identities of African descended Americans through the lens of Black culture.	Background listening before class discussion, or assign after a lecture on DTC genetic testing	https://inthosegenes.com/
peer-reviewed article	The Illusion of Inclusion — The "All of Us" Research Program and Indigenous Peoples' DNA	2020	Keolo Fox	2.5	Genetics		either	Indigenous geneticist (Native Hawaiian)	Bio on website (https://anthropology.ucsd.edu/people/faculty/faculty-profiles/Keolu%20Fox.html)	United States	A short written commentary by Dr. Fox on the "All of Us" NIH research programs, and how they may not be inclusive. This article is succinct but highlights many questions and considerations about indigenous genetics.	Reading for class discussion or as part of a online discussion	https://www.nejm.org/doi/pdf/10.1056/NEJMp1915987?articleTools=true

TAB: CLASSROOM RESOURCES - PLEASE SEE tinyurl.com/DACS-list

Peer-reviewed article	Exploring the potential of food forestry to assist in ecological restoration in North America and beyond	2017	Park, H., Turner, N., and Higgs, E. (2017). Exploring the potential of food forestry to assist in ecological restoration in North America and beyond. Restoration Ecology. 26: 284-293	10	Restoration Ecology	Urban Ecology	upper	Korean scientist	Thesis acknowledgements	Canada	Park et al. (2017) discuss incorporating food forestry in restoration ecology specifically towards urban settings. There's a good discussion on meeting needs of both people and conservation and how to integrate them successfully. There are good examples included as well as an overall description of practices.	This is a good paper for expanding restoration ecology into urban settings and discussing multiple goals. There are good figures that can be incorporated into lectures as well as combining both community health and needs with conservation goals.	https://onlinelibrary.wiley.com/doi/full/10.1111/rec.12576
peer-reviewed article	Promoting social and environmental justice to support indigenous partnerships in urban ecosystem restoration	2021	Hall, M. M., Wehi, P. M., Whaanga, H., Walker, E. T., Koia, J. H., & Wallace, K. J. (2021). Promoting social and environmental justice to support Indigenous partnerships in urban ecosystem restoration. Restoration Ecology, 29(1), e13305.	7	Restoration Ecology	Traditional Ecological Knowledge	either	Several Māori authors	J Koia interview (https://www.thekudos.org.nz/dr-jonni-koia/), H. Whaanga bio on website (https://www.waikato.ac.nz/fmis/about/staff/hemi)	Aotearoa New Zealand	Promotes partnerships with Indigenous communities from project inception and presents two successful case studies from Aotearoa New Zealand		https://onlinelibrary.wiley.com/doi/pdf/10.1111/rec.13305
Peer-reviewed article	Contributions of traditional knowledge to ecological restoration: Practices and applications	2015	Uprety, Y., Asselin, H., Bergeron, Y., Doyon, F., & Boucher, J. F. (2012). Contribution of traditional knowledge to ecological restoration: practices and applications. Ecoscience, 19(3), 225-237.	12	Restoration Ecology		either			Nepal	A review of the contributions of Indigenous people to restoration practice. Includes good background on what ecological restoration is.	A good intro into the topic for early in the semester - defines ecological restoration, talks about SER, but also discusses Indigenous contributions.	https://www.tandfonline.com/doi/abs/10.2980/19-3-3530
peer-reviewed article	A framework for enhancing ethical genomic research with Indigenous communities	2018	Claw et al (Indigenous researchers)	7	Responsible/Respectful Research Methods	Genetics	upper/instructor resource	Indigenous researchers			This article was the produced as part of the Summer internship for INdigenous peoples in Genomics (SING), and provides a framework for enhancing ethical genomic research with Indigenous communities. It's a great resource to read before (and after) initiating partnerships with indigenous communities.	Useful when planning research projects, to read as part of a ethics class. The paper has clear discussion points that could work for group activities or for a class discussion too.	https://www.nature.com/articles/s41467-018-05188-3.pdf
podcast	FreshEd #221: Indigenous Research Methodologies	2020	The FreshEd Podcast	34:24	Responsible/Respectful Research Methods		either	2 people interviewed. One is Wanka and Quechua, and I'm not sure of the other person's identity	Bio on faculty website		How can we think of indigenous knowledge systems as a paradigm for research methodology? With me are Elizabeth Sumida Huaman and Nathan Martin to discuss their new co-edited volume entitled Indigenous Knowledge Systems and Research Methodologies: Local Solutions and Global Opportunities.		https://soundcloud.com/freshed-podcast/221-sumida-huaman-martin
peer-reviewed article	Six ways to foster community-engaged research during times of societal crises	2020	Edwards, Hillary A., Dwyan Y. Monroe, and C. Daniel Mullins. "Six ways to foster community-engaged research during times of societal crises." (2020): 1101-1104.	3	Responsible/Respectful Research Methods		either				This paper provides a 10-step framework for continuous patient engagement. It considers both community and researcher perspectives to provide actionable considerations and strategies for community engagement. This paper is especially relevant in light of COVID-19 impacts.	This paper could be used for discussion in group settings (one group discusses and presents community perspectives, another group discusses and presents research perspectives)	https://www.ncbi.nlm.nih.gov/pmc/articles/PMC7668134/pdf/cer-09-1101.pdf
Peer-reviewed article	Indigenous Traditional Ecological Knowledge and Ocean Observing: A Review of Successful Partnerships	2021	Proulx M, Ross L, Macdonald C, Fitzsimmons S and Smit M (2021) Indigenous Traditional Ecological Knowledge and Ocean Observing: A Review of Successful Partnerships. Front. Mar. Sci. 8:703938. doi: 10.3389/fmars.2021.703938	17	Responsible/respectful Research Methods	Traditional Ecological Knowledge	Either	Nishnaabe-Scottish Kwe	https://ca.linkedin.com/in/maryjane-proulx-8606bb183	Canada	Shows example of successful integration of TEK and western knowledge based systems. They highlight relevant methodology that makes programs successful	I think that this would be particularly useful to have a large example of TEK/western programs	https://www.frontiersin.org/articles/10.3389/fmars.2021.703938/full
popular article	Five shifts to decolonise ecological science - or any field of knowledge	2021	Trisos, Auerbach, & Katti	3	Responsible/respectful Research Methods	Ecology/Intro Ecology	either	Middle author white from South Africa, last author an immigrant from India	ASM biography, author by middle author	South Africa	This is an accessible article written by the same authors as Trisos et al (2021) previous resource.	I think this would be a good reading for an lower-level or as a way to introduce these topics, and the peer-reviewed article (also included here) would be better for upper-level classes.	https://theconversation.com/five-shifts-to-decolonise-ecological-science-or-any-field-of-knowledge-161750
popular article	Keeping Indigenous Science out of a Colonial Mind (Interview with Dominque David-Chavez)	2019	Article by Kimberly M. S. Cartier for Eos	6	Responsible/respectful Research Methods	Environmental Science	either	Interviewee is Arawak Taíno	In linked source		A new working model could help scientists design and facilitate research that adheres to both scientific and cultural ethics standards when working with indigenous knowledge about climate and the environment.	This is an article that would be suitable for both lower- and upper-level classes, and promininetly features an Indigenous scientist and how she does research in collaboration with her community. It also shows how other models of research are extractive and harmful.	https://eos.org/articles/keeping-indigenous-science-knowledge-out-of-a-colonial-mold
website	Karuk Traditional Ecological Knowledge and the Need for Knowledge Sovereignty: Social, Cultural and Economic Impacts of Denied Access to Traditional Management	2016	Karuk Climate Change Projects		Intro Ecology	Traditional Ecological Knowledge	either	Karuk Tribe	in linked source	Karuk Tribe and United States	Website with different chapters which each could be used for a lesson topic. Tabs include the climate action plan, fire ecology, vulnerability assessments, and sovereignty. Each topic (Climate Vulnerability Assessment, Karuk TEK and Knowledge Sovereignty, and Retaining Knowledge Sovereignty) has multi-chapter readings and embedded videos.	I think that the webistes, chapters, and videos here are an accessible resource that highlights climate change science, traditional ecological knowledge, and integrates knowledge/research systems with discussions on sovereignty.I think that either single chapters/topics could be included in a course or working through multiple chapters throughout a course would be great.	https://karuktribeclimatechangeprojects.com/about/karuk-tek-knowledge-sovereignty/
popular article	Science Still Bears the Fingerprints of Colonialism	2018	Rohan Deb Roy		Intro Ecology	Intro Biology	either			UK	Modern Western science relied heavily on imperial colonialist structures to collect specimens and curate knowledge. This took the form of the use of the labor of enslaved people, the reliance on global imperial trade networks to collect specimens and objects, and the reliance on wealth accumulated through colonial exploits to fund scientific expeditions and endeavors	An accesible article to highlight the range of ways that scientific knowledge is connected to the world's prevailing power structures and problems (racism and colonialism). It provides both a good introduction to racist and colonial history of science and also modern iterations of these forces.	https://www.smithsonianmag.com/science-nature/science-bears-fingerprints-colonialism-180968709/
peer-reivewed article	Asian Americans: The Forgotten Minority in Ecology	2020	Sian Kou-Giesbrecht		Intro Ecology	DEIJ in STEM	either			USA	This article discusses the lack of Asian-American representation in the ecology field. The article goes on to state the case about why this lack of representation is at the detriment to the field and science education.	Discussion about the state of cultural diversity in the ecology field and why it is important to have representation, and in this specfic case, Asian-American respresentation.	https://esajournals.onlinelibrary.wiley.com/doi/10.1002/bes2.1696
popular article	How racism and classism affect natural ecosystems	2020	Jake M Robinson		Intro Ecology		either, probably lower			UK	Very accesible and short article introducing the concept that racism and classism shape natural ecosystems.	Great for getting early stage students thinking about the connections between people and nature and specifically the connections between racism and ecosystem function and structure. Includes discussion of Schell 2020, which is nice and connects it to other topics.	https://theconversation.com/how-racism-and-classism-affect-natural-ecosystems-144751
podcast	Healing the Land IS Healing Ourselves	2020	All My Relations podcast, Hosts Adrienne Keene and Matika Wilbur interviewing Kim Smith		Intro Ecology/Restoration Ecology	Environmental justice	either	hosts are Cherokee and Tulalip, guest is Dine	on All My Relations website	Cherokee and Tulalip Nations	The hosts talk with Kim Smith (Dine), who is a community organizer, citizen scientist, activist, water protector, entrepreneur, writer, gardener. Kim discusses how to understand how violence on the land is violence on our bodies, and that the inverse can also be true—healing the land is healing ourselves	Possibly useful for making storied connections between human and more-than-human overlapping ecologies	https://www.allmyrelationspodcast.com/podcast/episode/4bb3fff4/healing-the-land-is-healing-ourselves
peer reviewed article	Elevate, don't assimilate, to revolutionize the experience of scientists who are Black, Indigenous and people of color	2020	Halsey, S. J., Strickland, L. R., Scott-Richardson, M., Perrin-Stowe, T., & Massenburg, L. (2020). Elevate, don't assimilate, to revolutionize the experience of scientists who are Black, Indigenous and people of colour. Nature Ecology & Evolution, 4(10), 1291-1293.	2	DEIJ in STEM	Ecology and Evolution	either	lead author is Black	In resource	United States	The resource states, "As early-career Black women, we argue that encouraging assimilation is not enough to address systemic racism and outline suggestions for how minoritized individuals can not only survive, but thrive, in ecology and evolutionary biology."	This paper has some nice images that could be used in classes talking about the importance of diversity in ecological science and how to thrive as BIPOC in ecology.	https://www.nature.com/articles/s41559-020-01297-9
peer reviewed article	Anti-racist interventions to transform ecology, evolution, and conservation biology departments	2021	Cronin, M. R., Alonzo, S. H., Adamczak, S. K., Baker, D. N., Beltran, R. S., Borker, A. L., ... & Zavaleta, E. S. (2021). Anti-racist interventions to transform ecology, evolution and conservation biology departments. Nature Ecology & Evolution, 5(9), 1213-1223.	10	DEIJ in STEM	Conservation Biology	either	"This work was authored by a diverse group of EECB faculty members, staff and students of multiple racial and ethnic identities"	In resource	United States	"To spark restorative discussion and action in these areas, we summarize EECB's racist and ethnocentric histories, as well as current systemic problems that marginalize non-white groups. Finally, we present ways that EECB departments can collectively address shortcomings in equity and inclusion by implementing anti-racism" (interview by authors here)	Could be used in discussion of history of oppression within EECB disciplines and what we can do about it.	https://www.nature.com/articles/s41559-021-01522-z
peer reviewed article	An ecology of segregation	2020	Pickett, S. T., & Grove, J. M. (2020). An ecology of segregation.	1	DEIJ in STEM	Responsible/respectful Research Methods	upper	One of the authors is Black	In resource	United States	"the time has come for the science to more fully acknowledge the existence and impact of systemic racism, especially in the US. Acknowledging this fact in the various specialties within our discipline would complement the scholarship on environmental justice, and support the expanding efforts of ESA to be more racially diverse and inclusive."	This article could be used in a class about responsible research methods to discuss how racism could be included in ecological studies.	https://esajournals.onlinelibrary.wiley.com/doi/full/10.1002/fee.2279
peer-reviewed	Grizzly bear monitoring by the Heiltsuk people as a crucible for First Nation conservation practice	2014	Housty, W.G., Noson, A., Scoville, G.W., Boulanger, J., Jeo, R.M., Darimont, C.T. and Filardi, C.E., 2014. Grizzly bear monitoring by the Heiltsuk people as a crucible for First Nation conservation practice. Ecology and Society, 19(2).	12	Conservation Biology	Responsible/respectful Research Methods, Traditional Ecological Knowledge	upper&lower	Indigenous led study	see resource below	Canada	Research collaboration among academics, tribal government, provincial and federal government, resource managers, conservation practitioners, and community leaders supporting First Nation resource authority and stewardship. This study on bear genetics is "guided by principles from Gvi'ilas or customary law, this research methodology is coupled with Heiltsuk culture, enabling results of applied conservation science to involve and resonate with tribal leadership in ways that have eluded previous scientific endeavors. In this context, we discuss the effectiveness of research partnerships that, from the outset, create both scientific programs and integrated communities of action that can implement change."	Use in conjunction with the media release below for a discussion class (upper/lower level), or use as a specific case study for upper level clases. Has very useful tables for frameing conservations science withing indigenous principles to develop community centered conservation. Older study, but one of the better ones I've seen.	https://www.jstor.org/stable/pdf/26269572.pdf
press release	STUDY LED BY INDIGENOUS PEOPLE UNCOVERS GRIZZLY BEAR 'HIGHWAY'	2014	Kendra Snyder, Department of Communications, AMNH	1	Conservation Biology	Responsible/respectful Research Methods, Traditional Ecological Knowledge	upper&lower				Press release for previous study - use jointly?	This is a great press release and summary for the study above, could be used jointly - or as an introduction to encourage a deeper understanding of community centered conservation	https://www.amnh.org/content/download/76566/1461389/file/Heiltsuk%20grizzly%20bears.pdf
peer-reviewed	Indigenous knowledge and the shackles of wilderness	2021	Fletcher, M. S., Hamilton, R., Dressler, W., & Palmer, L. (2021). Indigenous knowledge and the shackles of wilderness. Proceedings of the National Academy of [illegible]	7	Conservation Biology	Traditional ecological knowledge	either	first author is Wiradjuri	author twitter (@theotheroad)	Australia	Provides a brief background on the concept of "wilderness", and argues that its a eurocentric, inappropriate, and dehumanizing construct. Includes case studies that show the importance of human interaction with the land. Solutions: Indigenous and community conservation areas must be legally recognized and supported to enable socially just, empowering, and sustainable conservation across scale.	Useful for a class covering the differences and ethical considerations between conservation and preservation. There's a news article that shares the highlights that would be more accessible for a lower level class. The peer-reviewed manuscript is also fairly short.	https://www.pnas.org/content/pnas/118/40/e2022218118.full.pdf

TAB: CLASSROOM RESOURCES - PLEASE SEE tinyurl.com/DACS-list

magazine article	How Conservation Became Colonialism: Indigenous people, not environmentalists, are the key to protecting the world's most precious ecosystems.	2018	Alexander Zaitchik		Conservation Biology		either				This article discusses the role of "green colonialism" in shaping conservation efforts and national park management in Ecuador. The story centers around Cayambe Coca National Park and discusses how the indigenous residents of the park are deprived of their fundamental rights and coerced into inequitable relationships with park officials and into conflict with illegal mining operations	This article would be useful for helping students see the ongoing effects of colonialism outside of the North American context.	https://foreignpolicy.com/2018/07/16/how-conservation-became-colonialism-environment-indigenous-people-ecuador-mining/
peer-reviewed	Safe fieldwork for at-risk individuals, their supervisors, and institutions	2021	Demery, A. J. C., & Pipkin, M. A. (2021). Safe fieldwork strategies for at-risk individuals, their supervisors and institutions. Nature Ecology & Evolution, 5(1), 5-9.	4	DEIJ in STEM	Ecology	either	A. Demery (Black), M. Pipkin (Black)	A. Demery - Cornell website; M Pipkin - Cornell Sun		"As a result of identity prejudice, certain individuals are more vulnerable to conflict and violence when they are in the field. It is paramount that all fieldworkers be informed of the risks some colleagues may face, so that they can define best practice together: here we recommend strategies to minimize risk for all individuals conducting fieldwork."	This article can be used to increase awareness of the unique risks for BIPOC and other at-risk individuals, as well as discussion for how we can make fieldwork more accessible. There is the recommendation to 'follow local laws' which we interpret to mean 'be aware of local laws and understand associated risks' as some identities are criminalized.	https://www.nature.com/articles/s41559-020-01328-5
peer-reviewed	Who are we? Highlighting nuances in Asian American experiences in ecology and evolutionary biology.	2021	Nguyen, K.H., Akiona, A. K., Chang, C. C., Chaudahry, V. B., Cheng, S. J., Johnson, S. M., Kahanamoku, S. S., Lee, A., de Leon Sanchez, E. E., Segui, L. M., & Tanner, R. L. (2021). Who are we? Highlighting nuances in Asian American experiences in ecology and evoluitonary biology. Ecological Society Bulletin, e01939.		DEIJ in STEM	Ecology	either	Some of the authors are Pacific Islanders - implied others are Asian American	Linked resource		"As ecologists and evolutionary biologists, it is important to recognize that issues surrounding AAPI do not exist in a vacuum and that these issues affect some of our students and colleagues in and outside of the classroom and lab."	Discussion of the unique barriers experienced by Asian American scientists and students.	https://esajournals.onlinelibrary.wiley.com/doi/full/10.1002/bes2.1939
peer-reviewed	Overcoming racism in the twin spheres of conservation science and practice	2021	Rudd, L. F., Allred, S., Bright Ross, J. G., Hare, D., Nkomo, M. N., Shanker, K., ... & Dávalos, A. (2021). Overcoming racism in the twin spheres of conservation science and practice. Proceedings of the Royal Society B, 288(1962), 20211871.		DEIJ in STEM	Conservation Biology	either	"We, the authors, are a diverse team representing, other than race, different ethnicities"	Linked resource		The authors "describe how the mutually reinforcing 'twin spheres' of conservation science and conservation practice perpetuate this systemic racism."	A great figure about how conservation science and practice supports racism. This paper could be used as a discussion piece about how institutions in conservation "systematically produce conservation graduates with partial and probematic concepttions of conservation's history and contemporary purposes"	https://royalsocietypublishing.org/doi/full/10.1098/rspb.2021.1871
peer-reviewed	Ecology and evolutionary biology must elevate BIPOC scholars	2021	Massey, Melanie Duc Bo, et al. "Ecology and evolutionary biology must elevate BIPOC scholars." Ecology Letters 24.5 (2021): 913-919.	5	DEIJ in STEM	Ecology	either	"Early-career BIPOC EEB researchers"	Linked resource	United States	"Black, Indigenous and people of colour (BIPOC) individuals are disproportionately impacted by the negative consequences of our ongoing environmental and climate crises, yet their valuable scientific voices are shockingly underrepresented within the fields of Ecology and Evolutionary Biology (EEB)."	This paper is a call to action, is short and accessible, and suggests specific steps that can be taken: anti-racism education and practice, increased fundign opportunities, integration of diverse cultural perspectives, and a community-minded shift.	https://onlinelibrary.wiley.com/doi/abs/10.1111/ele.13716
popular article	I'm an environmental journalist, but I never write about overpopulation. Here's why.	2018	Roberts, David. (2018) I'm an environmental journalist, but I never write about overpopulation. Here's why. Vox. November 29, 2018<br>Since you asked (many times).		Human Population Impacts	Wildlife/Fisheries Population Ecology	Instructor				This article is written by an environmental journalist and addresses several key problems surrounding the discourse of overpopulation. It introduces the idea that population size alone does not predict environmental impact and consumption and technology must be taken into account.	Discussions of population ecology naturally lend themselves to students thinking about exponential growth of human populations and their impact on the planet. Many renowned ecologists, including Paul Ehrlich, have raised their concerns about the perceived problem. This article could be a useful way of addressing misconceptions surrounding overpopulation and addressing the questions students may have head-on. It connects fears of overpopulation to the undercurrents of racism and xenophobia that often taint such discussions. One discussion participant has used this in lectures and students tend to still latch on to overpopulation as the driving factor from some of the paragraphs in the middle. Given some student difficulty in extracting the information, we recommend using this for figures and as an instructor resource to present the concepts of the interactions of overpopulation and affluence, but to not use this as an assigned reading.	https://www.vox.com/energy-and-environment/2017/9/26/16356524/the-population-question
video	Why Overpopulation Isn't the Problem You Think It Is	2021	Seeker [Youtube Channel] (2021). Why Overpopulation Isn't the Problem You Think It Is. January 16, 2021 https://www.youtube.com/watch?v=sQz95D1LgyY		Human Population Impacts	Wildlife/Fisheries Population Ecology	Either	Unclear		Unknown	Short video discussion the topic of overpopulation and the importance of considering both affluence/consumption and technology alongside population when evaluating the impact of humans on the environment.	Many students in population ecology courses may wish to apply what they've learned about population growth in wildlife to human populations. Human overpopulation is a popular topic of discussion in environmental circles. This video lays out common misconceptions about the population control argument. While it does not explicity address racism or xenophobia, it would be a great starting point for such discussions, especially when paired with an article that describes such issues explicitly.	https://www.youtube.com/watch?v=sQz95D1LgyY
Peer-reviewed article	Age- and sex-specific survival of the Gunnison's prairie dog (cynomys gunnisoni)	2022	Farid, R. H., Karelus, D. L., & Hull, V. (2022). Age-and sex-specific survival of the Gunnison's prairie dog (Cynomys gunnisoni). Ecosphere, 13(3), e3937.		Wildlife/Fisheries Population Ecology	Wildlife Management	Upper	First author is Black	https://www.esa.org/events/the-esa-weekly-water-cooler/black-ecologists-shaping-the-science-of-ecology/		Cature-mark-recapture analysis of Gunison's prairie dogs, implementing the following models: age-cohort Cormack-Jolly-Seber, multistate mark-recapture models, postbreeding matrix ppulation model, elasticity analysis	Example / case study for class covering any of the methods used in this paper	https://esajournals.onlinelibrary.wiley.com/doi/pdfdirect/10.1002/ecs2.3937
Peer-reviewed	Diverse knowledge systems reveal social–ecological dynamics that inform species conservation status	2018	Lee, L. C., Thorley, J., Watson, J., Reid, M., & Salomon, A. K. (2019). Diverse knowledge systems reveal social–ecological dynamics that inform species conservation status. Conservation Letters, 12(2), e12613.		Wildlife/Fisheries Population Ecology	Traditional ecological knowledge	Upper				"We synthesized zooarchaeological, historical, traditional, and western science<br>knowledge to document changes in relative abundance of key species in Canada's<br>northern abalone social–ecological system (SES) from the Holocene to present."		https://conbio.onlinelibrary.wiley.com/doi/pdfdirect/10.1111/conl.12613
Peer-reviewed	Comparison of reintroduction and enhancement effects on metapopulation viability	2015	Halsey SJ, Bell TJ, McEachern K, Pavlovic N. 2015. Comparison of reintroduction and enhancement effects on metapopulation viability. Restoration Ecology. 23(4):375-384.		Plant Population Ecology	Restoration Ecology	Upper	First Author Black	I am she	USA			https://onlinelibrary.wiley.com/doi/10.1111/rec.12191
Peer-reviewed	Indigenous peoples and salmon stewardship: a critical relationship	2020	Carothers, C., J. Black, S. J. Langdon, R. Donkersloot, D. Ringer, J. Coleman, E. R. Gavenus, W. Justin, M. Williams, F. Christiansen, C. Stevens, B. Woods, S. Clark, P. M. Clay, L. Mack, J. Raymond-Yakoubian, A. Akall'eq Sanders, B. L. Stevens, and A. Whiting. 2021. Indigenous peoples and salmon stewardship: a critical relationship. Ecology and Society 26(1):16. https://doi.org/10.5751/ ES-11972-260116		Wildlife/Fisheries Population Ecology	Traditional ecological knowledge	Upper and lower	Author list includes indigenous scholars - also includes a positionality statement	positionality statement in the article	USA	This article is provides a synthesis of what is known about the diverse human-salmon relationships, with specific reference to the connection among Alaskan Indiginous communities and salmon. "Two important socio-cultural dimensions of salmon–people systems emerged from this synthesis as fundamentally important but largely invisible outside of Indigenous communities and the social science disciplines that work closely with these communities: (1) the deep relationships between Indigenous Peoples and salmon and (2) the pronounced inequities that threaten these relationships and stewardship systems."	There are many ways that I envision this article be used in the classroom. First, I feel like it is a very valuable source for framing the impotance of fishing (in this case specifically salmon) as part of Indigenous cultures - I therefore see this as a background reading/at home reading. I also see how this article can be used in small discussion groups to delve deeper into certain aspects. For example, discussions can be devided per topic (relationship between peoples and salmon, inequities in the salmon-people system), or could discuss how relationship are unqiuely deep in different tribal cultures.	https://www.ecologyandsociety.org/issues/view.php?sf=139
Peer-reviewed	Congruence of local ecological knowledge (LEK)-based methods and line-transect surveys in estimating wildlife abundance in tropical forests	2022	Braga-Pereira, F., Morcatty, T. Q., El Bizri, H. R., Tavares, A. S., Mere-Roncal, C., González-Crespo, C., ... Mayor, P. (2022). Congruence of local ecological knowledge (LEK)-based methods and line-transect surveys in estimating wildlife abundance in tropical forests. Methods in Ecology and Evolution, 13(3), 743–756. doi:10.1111/2041-210X.13773	14	Wildlife/Fisheries Population Ecology	Local Ecological Knowledge	Upper			Brazil, Spain, USA, Peru, UK	This paper connects LEK data collection to line-survey abundance estimates. It highlights the benefit of LEK especially for hard to detect species	This paper would be good for expansion of an existing population ecology course that does not have lots of TEK, LEK, or diversified pedagogy. It pushes against the concept that field sampling approaches produce the best data, and increases the involvement of local communities for stronger data. Particularly highlights the biases common in western ecological methods	https://besjournals.onlinelibrary.wiley.com/doi/full/10.1111/2041-210X.13773?campaign=wolearlyview

TAB: CLASSROOM RESOURCES - PLEASE SEE tinyurl.com/DACS-list

ID	Title	Source	Creator Identity Notes	Creator Identity Source Information	Topic	Link
IR8	Guidelines for Respecting Cultural Knowledge	Assembly of Alaska Native Educators, published bye the Alaska Native Knowledge Network	Authors are Alaska Native		Race and Ethnicity	https://drive.google.com/file/d/0B-SWQftFc_U1cDRGVlhLdmd6M1k/view
IR10	Effective Teaching is Antiracist Teaching	Brown University - The Harriet W. Sheridan Center for Teaching and Learning			Race and Ethnicity	https://www.brown.edu/sheridan/teaching-learning-resources/inclusive-teaching/effective-teaching-anti-racist-teaching
IR13	The Anti-Racist Discussion Pedagogy	packback	First author is Chinese/Latinx; The authorship team was diverse and composed of African American and Chinese/Latinx scholars	Provided by author	Race and Ethnicity	https://www.packback.co/resources/anti-racist-discussion-pedagogy-guide/confirmation/#contact-form-12635
IR24	Our Younger Selves: QPOC Student Affairs Professionals Supporting QPOC Students	Vijay Kanagala & Steven Thurston Oliver (2019) Our Younger Selves: QPOC Student Affairs Professionals Supporting QPOC Students, Equity & Excellence in Education, 52:4, 409-423, DOI: 10.1080/10665684.2019.1705204			Gender and Sexuality	https://www.tandfonline.com/doi/abs/10.1080/10665684.2019.1705204?journalCode=ueee20
IR31	"Best practices for serving LGBTQ Students"	Southern Poverty Law Center			Gender and Sexuality	https://www.learningforjustice.org/sites/default/files/2018-09/TT-LGBTQ-Best-Practices-Guide.pdf
IR32	Creating Accessible Learning Environments	Thurber, A., & Bandy, J. (2018). Creating Accessible Learning Environments. Vanderbilt University Center for Teaching.			Disability	https://cft.vanderbilt.edu/guides-sub-pages/creating-accessible-learning-environments/
IR34	Theorizing race and settler colonialism within U.S. sociology	McKay, D. L., Vinyeta, K., & Norgaard, K. M. (2020). Theorizing race and settler colonialism within US sociology. Sociology Compass, 14(9), e12821.	K Norgaard: non-Native sociologist	Bio in The Conversation	Settler-Colonialism & Race	https://onlinelibrary.wiley.com/doi/abs/10.1111/soc4.12821?casa_token=W5rUrdz6LlQAAAAA:MSQrrsCs0MkMqSfu6VvL1MnWHjZsTx9dkzOxlg52Imf3j91gZhbmprsP9tcJ8u17b8xMeNTnomb_7lmq
IR35	Reframing anti-colonial theory for the diasporic context	Simmons, M., & Sefa Dei, G. J. (2012). Reframing anti-colonial theory for the diasporic context.	Dei: Ghanian-born	Dei: University Bio	Settler-Colonialism	https://scholar.google.com/scholar?hl=en&as_sdt=0%2C15&q=Reframing+anti-colonial+theory+for+the+diasporic+context&btnG=
IR36	Decolonization is not a metaphor (MUST READ)	Tuck, E., & Yang, K. W. (2012). Decolonization is not a metaphor. Decolonization: Indigeneity, education & society, 1(1).	Tuck: Unangax̂ and is an enrolled member of the Aleut Community of St. Paul Island, Alaska; Yang: non-Native	Tuck: Bio on website Yang: linked resource	Decolonization	https://jps.library.utoronto.ca/index.php/des/article/view/18630
IR37	This land is Our land? This land is Your Land: The Decolonizing Journeys of White Outdoor Environmental Educators	Root, E. (2010). This Land is Our Land? This Land is Your Land: The Decolonizing Journeys of White Outdoor Environmental Educators. Canadian Journal of Environmental Education, 15, 103-119.	white	Linked resource	Decolonization	https://eric.ed.gov/?id=EJ942807
IR38	Asian Americans: The Forgotten Minority in Ecology	Sian Kou-Giesbrecht				https://esajournals.onlinelibrary.wiley.com/doi/10.1002/bes2.1696
IR40	Anti-racist interventions to transform ecology, evolution, and conservation biology departments	Cronin, M. R., Alonzo, S. H., Adamczak, S. K., Baker, D. N., Beltran, R. S., Borker, A. L., ... & Zavaleta, E. S. (2021). Anti-racist interventions to transform ecology, evolution and conservation biology departments. Nature Ecology & Evolution, 5(9), 1213-1223.	"This work was authored by a diverse group of EECB faculty members, staff and students of multiple racial and ethnic identities"	In resource		https://www.nature.com/articles/s41559-021-01522-z

TAB: INSTRUCTOR RESOURCES

Name	Topic 1	Topic 2	Major contributions	Self-described identity	Notes
Dr. Wangari Maathai (1940-2011)	Conservation Biology	Environmental Science	Green Belt Movement aimed at reforestation in Kenya and empowering women.	Kenyan	Nobel Peace Prize Recipient
Chico Mendes (1944-1988)	Conservation Biology	Sustainable Use	Amazon conservation, extractive reserves	Brazilian	"Patron of Brazilian Environment"
Dr. Robin Wall Kimmerer	Botany	Restoration Ecology	Proponent of integrating TEK into biology/ecology and environmental science	Potawotami	Author "Braiding Sweetgrass"
Dr. Mamie Parker	Conservation Biology	Fisheries	Led national conservation projects, created National Fish Habitat Action Partnership	Black	Received numerous awards over the course of her career; excellent profile here: https://uapbnews.wordpress.com/2020/04/10/dr-mamie-parker-uapb-alumna-reflects-on-30-year-career-as-pioneer-in-conservation/
Dr. Scott Edwards	Ornithology		Harvard professor, studies evolution, including speciation, beiogeography, evolution of the genome, and adaptation	Black	In addition to research, Dr. Edwards biked across the country to raise awareness for BLM and Black Birders week
W.E.B. Du Bois (1868-1963)	Rural sociology	Agriculture	"Du Bois' empirical research... provides evidence that Du Bois was among the first American sociologists to conduct empirical agrarian analyses and case studies of rural communities"	Black	Found in Jakubek & Wood (2018), and Bhambra (2014)
Benjamin Banneker (1793-1806)	Entomology	Naturalist	documented cicadas for 50 years in the early 1800s, discovering the 17-year cycle	Black	https://www.npr.org/2021/05/11/995898681/a-black-scientist-was-an-early-cicada-researcher-his-work-has-been-mostly-overlo?utm_campaign=storyshare&utm_source=twitter.com&utm_medium=social
Harriet Tubman (1822-1913)	Black liberation	Naturalist	Underground Railroad conductor using close knowledge and associations with nature to succeed	Black	https://www.audubon.org/news/harriet-tubman-unsung-naturalist-used-owl-calls-signal-underground-railroad
Matthew Henson (1866-1955)	Exploration		First person or one of the first people to the north pole	Black	https://en.wikipedia.org/wiki/Matthew_Henson ; https://www.youtube.com/watch?v=HNJCI3EtTtE
James Beckwourth (1798-1866)	Wildlife Management		Fur Trader, explorer, person to add when discussing early fur industry and western US	Black, Crow Nation	https://en.wikipedia.org/wiki/James_Beckwourth
Colonel Charles Young (1864-1922)	Conservation Biology		First Black superintendent of a National Park (1903) - Sequoia and general Grant national parks; several accomplishments during his leadership	Black	https://www.nps.gov/chyo/learn/historyculture/colonel-charles-young.htm
MaVynee Betsch (1935-2005)	Conservation Biology		40 years of activism for beach restoration and preservation	Black	https://www.smithsonianmag.com/history/beach-lady-84237022/; https://vault.sierraclub.org/sierra/200509/madame_butterfly.asp
Dorceta Taylor	Environmental Justice	Sustainability	Sociologist; relevant work for several environmental social science fields	Black	https://en.wikipedia.org/wiki/Dorceta_Taylor
J. Drew Lanham	Ornithology	Nature Writing	Author, poet, and ornithologist. Currently and endowed professor at Clemson University where he studies wildlife conservation and ecology. He has produced several collections of poetry and non-fiction creative writing on the topic of the environment, conservation, and nature. Recent popular article: https://orionmagazine.org/article/9-rules-for-the-black-birdwatcher/	Black	https://gardenandgun.com/feature/drew-lanham-birding/
John C. Robinson (1903-1954)	Ornithology	Birding	Wildlife biologist and ornithologist, author of Birding is For Everyone: Encouraging People of Color to Become Birdwatchers	Black	https://www.onmymountain.com
Dr. Christopher Schell	Urban Ecology	Environmental Justice	Leading important discussions on the impacts of systemic racism on urban ecosystems. Also conducts research on human-wildlife conflict in cities.	Black	https://ourenvironment.berkeley.edu/users/1757114
Dr. Ingrid Waldron	Human Health and the Environment	Environmental Justice	Author of the book "There's Something in the Water," which has been made into a Netflix documentary. Researches impacts of environmental racism on health. Founder and Director of the Environmental Noxiousness, Racial Inequities and Community Health Project.	Black	https://experts.mcmaster.ca/display/waldroni
Dudley Edmondson	Conservation Biology	Nature Writing	Author of the book Black and Brown Faces in America's Wild Places; also an amazing photographer and public speaker	Black	https://dudleyedmondson.com
Dr. Paula Kahumbu	Conservation Biology	Endangered Species Protection	Paula Kahumbu is a wildlife conservationist and chief executive officer of WildlifeDirect. She is best known as a campaigner for elephants and wildlife, spearheading the Hands Off Our Elephants Campaign, which was launched in 2014 with Kenyan First Lady Margaret Kenyatta		https://paulakahumbu.org/about/
Makoma Lekalakala	Environmental Justice	Sustainable use	Makoma Lekalakala is a South African activist who is the director of the Johannesburg branch of Earthlife Africa, an NPO that seeks a better life for all people without exploiting other people or degrading their environment. They encourage and support individuals, businesses and industries to reduce pollution, minimise waste and protect natural resources.	Black (South African)	https://earthlife.org.za
Dr. Godwell Nhamo	Sustainable Use	Environmental Science/Climate Change	Godwell Nhamo is a Full Professor and Exxaro Chair in Business and Climate Change at the University of South Africa (UNISA). He has published widely in the areas of Climate Change and Governance, Green Economy and Sustainable Development, including 6 books (5 edited) and over 70 journal articles.	Black (South African)	https://www.researchgate.net/profile/Godwell-Nhamo
Kaddu Sebunya	Conservation Biology	Endangered Species Protection	As African Wildlife Foundation's CEO, Kaddu rallies the continent's elite to lead the fight against the destruction of valuable habitats and wildlife. He believes that it is time for African voices and networks to re-imagine the conservation narrative.	Black	https://www.conservation-strategy.org/profile/kaddu-kiwe-sebunya
Belise Kariza	Conservation Biology	Endangered Species Protection	African Wildlife Foundation's Country Director, Rwanda - Great advocate for women in wildlife conservation science in Africa - see article link	Black	https://www.awf.org/news/seat-table-women-have-say-wildlife-conservation
Dr. Raman Sukumar	Ecology	Conservation Biology	Career focused on ecology of Asian elephants and human-wildlife conflict. Founded Asian Nature Conservation Foundation a trust that carries out projects to protect elephants and their habitats. Also, much of his research is focused climate change.	Indian	http://ces.iisc.ernet.in/new/?q=user/28
Dr. Champika Ellawala Kankanamge	Restoration Ecology	Environmental engineering	Career focus on restoring degraded aquatic environment and reducing invasive aquatic species. Monitors aquatic species population levels including species relied on by local communities for sustenance.	Sri Lankan	chrome-extension://efaidnbmnnnibpcajpcglclefindmkaj/viewer.html?pdfurl=https%3A%2F%2Fowsd.net%2Fsites%2Fdefault%2Ffiles%2FReference%2520Sheet%2520-%2520Ellawalla.pdf&clen=484769&chunk=true
Dr. Ernest Everett Just (1883-1941)	Embryology	Marine Biology	Studied cells in Oceans, wrote "The Biology of the Cell Surface"; sometimes called "TheForgotten Father of Epigenetics"	Black	https://www.google.com/search?q=Black+evolutionary+biologist&sxsrf=APq-WBsZ6JN_goW0CUNzcMm8fz2a-EvsFQ%3A1649687716156&ei=pDxUYtqXCY24tQa075XwDA&ved=0ahUKEwja-b7SnYz3AhUNXM0KHbR3Bc4Q4dUDCA4&uact=5&oq=Black+evolutionary+biologist&gs_lcp=Cgdnd3Mtd2l6EAMyBQgAEIAEMgUIABCGAzIFCAAQhgMyBQgAEIYDMgUIABCGAzIFCAAQhgM6BAgjECc6BAgAEEM6BQgAEJECOgcILhDUAhBDOgoIABCABBCHAhAUOggILhCABBCxAzoLCAAQgAQQsQMQgwE6CAgAEIAEELEDOgsILhCABBDHARCvAToGCAAQFhAeOggIABAWEAoQHkoECEEYAEoECEYYAFAAWNQbYLQcaABwAXgBgAHzAYgBpBaSAQYxOC44LjGYAQCgAQHAAQE&sclient=gws-wiz
Dr. Steward Pickett	Ecology	Botany	First Black ESA President, founding director of the Baltimore Ecosystem STudy, and important in urban sustainability.	Black	https://www.esa.org/events/the-esa-weekly-water-cooler/black-ecologists-shaping-the-science-of-ecology/
Gladys West	Math	GIS	Created an accurate model fo the Earth which was used as the foundation for the creation of the Global Positioning System (GPS). Earned a PhD at age 70	Black	https://www.pbs.org/education/blog/ten-black-scientists-that-science-teachers-should-know-about-and-free-resources; https://www.cbc.ca/radio/quirks/black-scientists-history-1.5918964
George Washington Carver (1861-1943)	Agriculture		Innovator of agricultural science; he focused a lot of his time on developing uses for teh peanut, including soap, face powder, mayonaise, shampoo, metal polish, and adhesives	Blak	https://www.famousscientists.org/15-famous-black-scientists-in-history/
Charles Henry Turner (1867-1923)	Biology	Neurology & Animal Behavior	Researcher in animal cognition, first Black scientist to be published in Science. Discovered that insects have the capacity to hear, that they learn by trial and error, and that bees use olfactory and visual cues to find nectar. Published 71 papers in his lifetime, including 3 in Science, even though he was not allowed to work at a university (he became a high school teacher at an all-black high school)	Black	https://www.cbc.ca/radio/quirks/black-scientists-history-1.5918964
Albert Baez (1912-2007)	Physics		Co-invented the x-ray reflection microsoft which allowed scientists to examine living cells. Also a pacifist during the cold War - refused defense industry positions.	Mexican-American	https://gladstone.org/news/spotlighting-latinx-scientists
Ynes Mexia (1870-1938)	Botany		Discovered two new plant genera and 500 new plant species (and didn't start collecting plants until she was 51!).	Mexican-American	https://gladstone.org/news/spotlighting-latinx-scientists
Multiple	Environmental Science		8 BIPOC Environmentalists Your History Textbooks Probably Left Out (That You Should Really Know!)	BIPOC	https://acespace.org/2020/09/14/8-bipoc-environmentalists/
Multiple	Environmental Science		10 BIPIOC Environmental Authors	BIPOC	https://wastefree.earth/10-bipoc-environmental-authors/
Multiple	Environmental Science		Celebrating Black Environmentalists during Black History Month	Black	https://sfenvironment.org/article/celebrating-black-environmentalists-during-black-history-month

TAB: PEOPLE TO KNOW

Name	Known for?	Article Title	Source/Creator	Year Created	Link
John Muir (1838-1940)	"Father of National Parks"	"Sierra Club Says It Must Confront the Racism of John Muir"	New York Times	2020	https://www.nytimes.com/2020/07/22/us/sierra-club-john-muir.html
John James Audubon (1785-1851)	Ornithology	"The Myth of John James Audubon"	Audbon.org	2020	https://www.audubon.org/news/the-myth-john-james-audubon
John James Audubon (1785-1851)	Ornithology	"What Do We Do About John James Audubon?"	audbon.org		https://www.audubon.org/magazine/spring-2021/what-do-we-do-about-john-james-audubon
William Hornaday	Widlife conservation / Bronx Zoo	"The Most Defiant Devil" (Book Review)	R. M. Wilson	2014	https://www.tandfonline.com/doi/full/10.1080/2325548X.2014.901856
William Hornaday	Widlife conservation / Bronx Zoo	"Decolonizing conservationist hero narratives: a critical genealogy of William T. Hornaday and colonial conservation rhetorics"	Marouf Arif Hasian Jr & S. Marek Muller (2019): Decolonizing conservationist hero narratives: a critical genealogy of William T. Hornaday and colonial conservation rhetorics, Atlantic Journal of Communication, DOI: 10.1080/15456870.2019.1624543	2019	https://www.tandfonline.com/doi/abs/10.1080/15456870.2019.1624543
Garrett Hardin	Environmental science "tragedy of the commons	"The tragedy of the tragedy of the commons"	Scientific American	2019	https://blogs.scientificamerican.com/voices/the-tragedy-of-the-tragedy-of-the-commons/
James Watson	"Father of Genetics" - assisted with discover of double helix	"James Watson Had a Chance to Salvage Hist Reuptation on RAce. He Made Things Worse"	The New York Times	2020	https://www.nytimes.com/2019/01/01/science/watson-dna-genetics-race.html
Louis Agassiz	Geological and Biological contributions	Louis Agassiz	Harvard	2021	https://eps.harvard.edu/louis-agassiz
Louis Agassiz	Geological and Biological contributions	Harvard Profits From Photos Of Slaves, Lawsuit Claims	NPR	2019	https://www.npr.org/2019/03/21/705382289/harvard-profits-from-photos-of-slaves-lawsuit-claims
EO Wilson	Biodiversity	The Complicated Legacy of E. O. Wilson	Scientific American	2021	https://www.scientificamerican.com/article/the-complicated-legacy-of-e-o-wilson/
EO Wilson	Biodiversity	The Last Refuge of Scoundrels	Science for the People	2022	https://magazine.scienceforthepeople.org/online/the-last-refuge-of-scoundrels/
EO Wilson	Biodiversity	Ideology as Biology	The New York Review	2022	https://www.nybooks.com/daily/2022/02/05/ideology-as-biology/
Garrett Hardin	Environmental science "tragedy of the commons"	Garrett Hardin	Southern Poverty Law Center		https://www.splcenter.org/fighting-hate/extremist-files/individual/garrett-hardin
Henry Harpending	Human Evolution	Henry Harpending	Southern Poverty Law Center		https://www.splcenter.org/fighting-hate/extremist-files/individual/henry-harpending
Richard Lynn	Psychology	Richard Lynn	Southern Poverty Law Center		https://www.splcenter.org/fighting-hate/extremist-files/individual/richard-lynn
William Shockley	Electronics	William Shockley	Southern Poverty Law Center		https://www.splcenter.org/fighting-hate/extremist-files/individual/william-shockley
Roger Pearson	Anthropologist	Roger Pearson	Southern Poverty Law Center		https://www.splcenter.org/fighting-hate/extremist-files/individual/roger-pearson
Linda Gottfredson	Education Psychologist	Linda Gottfredson	Southern Poverty Law Center		https://www.splcenter.org/fighting-hate/extremist-files/individual/linda-gottfredson
Michael Levin	Philosophy	Michael Levin	Southern Poverty Law Center		https://www.splcenter.org/fighting-hate/extremist-files/individual/michael-levin
Raymond Cattell	Psychology	Raymond Cattell	Southern Poverty Law Center		https://www.splcenter.org/fighting-hate/extremist-files/individual/raymond-cattell
Arthur Jensen	Education Psychologist	Arthur Jensen	Southern Poverty Law Center		https://www.splcenter.org/fighting-hate/extremist-files/individual/arthur-jensen
Charles Murray	Psychologist, author of 'the Bell Curve'	Charles Murray	Southern Poverty Law Center		https://www.splcenter.org/fighting-hate/extremist-files/individual/charles-murray
EO Wilson	Biodiversity	When the Hagiography Stops and the Truth-Telling Begins: The Legacy of E.O. Wilson	Michael Balter (Journalist)	2022	https://michaelbalter.substack.com/p/when-the-hagiography-stops-and-the

TAB: POTENTIALLY PROBLEMATIC FIGURES

	@handle (IG = Instagram; Twitter)	Platform	Brief Description	Direct Link Insta:	Direct Link Twitter:
1	@unlikelyhikers	Instagram	Unlikely Hikers is a diverse, anti-racist, body-liberating outdoor community featuring the underrepresented outdoorsperson.	https://www.instagram.com/unlikelyhikers/	
2	@hownottotravellikeabasicbitch	Instagram	Destination-specific advice on responsible travel, personal essays about traveling as people of color, and humorous rants about oblivious tourist practices—like thoughtless geotagging or only staying in resorts where a visitor won't interact with any real culture.	https://www.instagram.com/hownottotravellikeabasicbitch/	
3	@latinooutdoors	Instagram	Celebrating Latinx use of outdoor spaces	https://www.instagram.com/latinooutdoors/	
4	@vanessanakate1(IG), @vanessa_vash (Twitter)	Instagram and Twitter	Vanessa Nakate promotes environmental activism and organization. Vanessa runs many community service projects	https://www.instagram.com/vanessanakate1/	https://twitter.com/vanessa_vash?s=20
5	@mikaelaloach	Instagram and Twitter	Climate justice & antiracism activist. Has cool podcast: @theyikespodcast (IG)	https://www.instagram.com/mikaelaloach/	https://twitter.com/mikaelaloach?s=20
6	@greengirlleah	Instagram	Activist + eco-communicator - Council member for an awesome group (https://www.intersectionalenvironmentalist.com)	https://www.instagram.com/greengirlleah/	
7	@indigenousclimateaction	Instagram	Indigenous-led organization working to inspire and support Indigenous-led climate action	https://www.instagram.com/indigenousclimateaction/	
8	@queernature	Instagram	The description: decolonial queer ancestral futurism.apocalyptic ecology. interspecies relations. place-based skills. mysticism. ecophilosophy		
9	@n8ture_al	Twitter and Instagram	Alex Troutman is young Black Biologist sharing his love for Animals,Food,&Adventure #blackandstem #wildlife,#ConservationScientist #outdoorsman #blackscientists	https://www.instagram.com/p/B9LbuhigxTi/?igshid=gc63g3xsn98w	https://twitter.com/n8ture_al?s=20
10	@MozzieFhal	Twitter	Fallon Ware-Gilmore is a Public Health officer/ PhD student at Penn State studying med entomology. Fallon researches the impact that global climate change will have on the assemblage of infectious diseases, in particular, viruses transmitted by mosquitoes. #BlackInEnto #rollcall		https://twitter.com/MozzieFhal?s=20
11	@drdre4000	Instagram	This is the account of Dr. Andre K Isaacs. He showcases inclusive lab culture though comical video.	https://www.instagram.com/drdre4000/	
12	@BlackInGenetics	Twitter	This account highlights Black voices in genetics		https://twitter.com/BlackInGenetics
13	@BlkMammalogists	Twitter	This account highlights Black voices in mammalogy		https://twitter.com/BlkMammalogists
14	@BlackInEnviron	Twitter	This account highlights Black voices in environmental sciences		https://twitter.com/BlackInEnviron
15	@BlackAFinSTEM	Twitter	This account highlights Black voices in STEM		https://twitter.com/BlackAFinSTEM
16	@BioDiverseFest	Twitter	This account hosts diversity talks every October, highlighting positive representation and inclusion of minority identities in biology and conservation.		https://twitter.com/BioDiverseFest

TAB: DIVERSIFY YOUR SOCIAL MEDIA!
