# Supplementary material for: “Where do I even start?” Recommendations for faculty diversifying syllabi in ecology, evolution, and the life sciences
Source: Ecol Evol. 2023 Jan 3;13(1):e9719. doi: 10.1002/ece3.9719 (PMC9810791; doi:10.1002/ece3.9719)
Supplement: Supplementary file 3 — File S3 [file ECE3-13-e9719-s002.pdf]

Supplementary File 3: Combined land acknowledgement

Lands and waters are not as fixed, linear, and singular as "modern" colonial maps represent, and dismantling these Western conceptions of land is not simple. With that in mind, we would like to offer an acknowledgement of the Indigenous Peoples who practice/d culture, ceremony, and life on this sacred and ancestral ecology of lands and waters we occupy in the geographic areas colonially known as “Missouri", “Illinois”, “Indiana”, “Wisconsin”, “Colorado”, and “Pennsylvania.”

- ***University of Missouri:*** Wahzhazhe (Osage) people have some of the oldest and longest ties to this area and identify home lands and waters they belong to here in "Missouri." Ancestral Ponca, U-Mo'n-Ho'n (Omaha), and Kanza (Kaw) nations also have longstanding relationships with "Missouri." There are numerous other nations who were/are impacted by removals, reservations, and colonial borders in this area, including Nutachi (Missouria), Jiwere (Otoe), Othâkîwa/Thâkîwa (Sac and Fox), and Báxoǰe (Ioway) peoples. *This statement was created by MH.*
- ***University of Illinois at Urbana-Champaign:*** We would like to recognize and acknowledge that we live and work on the lands of the Peoria, Kaskaskia, Piankashaw, Wea, Miami, Mascoutin, Odawa, Sauk, Mesquaki, Kickapoo, Potawatomi, Ojibwe, and Chickasaw Nations. These lands were the traditional territory of these Native Nations prior to their forced removal; these lands continue to carry the stories of these Nations and their struggles for survival and identity. As a land-grant institution, the University of Illinois has a particular responsibility to acknowledge the peoples of these lands, as well as the histories of dispossession that have allowed for the growth of this institution for the past 150 years. *This statement was adapted from the UIUC Native American House.*

- ***Earlham College:*** We acknowledge that the Land we live and work on occupies the ancestral and contemporary Lands of the Delaware, Shawnee, and Miami tribes. In 1795, the U.S. government took much of Ohio and a narrow strip of Indiana (including Richmond, IN) through the Greenville Treaty. This was the westernmost white settlement at the time. The State of Indiana, Indiana colleges, and Indiana residents have benefitted from the forced removal of these people, many of whom now live in Oklahoma and Kansas. We would also like to acknowledge the ongoing presence of the Pokagon and Miami in Indiana. This statement does not absolve privilege or diminish structures of violence, and we recognize that ongoing commitments to Indigenous communities in Indiana must follow. *This statement was created by JJC.*
- ***University of Wisconsin-Madison:*** We would like to acknowledge that the land the University of Wisconsin-Madison occupies is the ancestral home of the Ho-Chunk Nation, who have called this land Teejop (day-JOPE) since time immemorial. In the first treaty following the Indian Removal Act in 1830, the state government forcibly removed the Ho-Chunk from their home in 1832. In the decades that followed, the federal and state government sought to completely remove the Ho-Chunk from Wisconsin. Despite these attempts, many Ho-Chunk people continued to return to their home in present-day Wisconsin. We acknowledge the circumstances that led to the forced removal of the Ho-Chunk people, and honor their history of resistance and resilience. The Ho-Chunk Nation and the other eleven First Nations residing in the boundaries of present-day Wisconsin remain vibrant and strong. *Modified by TPS from:UW-Madison Prevention Research Center.*

- ***Temple University:*** We recognize and acknowledge that Temple University stands on the Indigenous territory known as "Lenapehoking," the traditional homelands of the Lenape, also called Lenni-Lenape or Delaware Indians. These are the people who, during the 1680s, negotiated with William Penn to facilitate the founding of the colony of Pennsylvania. Their descendants today include the Delaware Tribe and Delaware Nation of Oklahoma; the Nanticoke Lenni-Lenape, Ramapough Lenape, and Powhatan Renape of New Jersey; and the Munsee Delaware of Ontario. *Modified by TMS from: UPenn Association of Native Alumni.*
- ***University of Colorado Boulder:*** We acknowledge that the University sits upon land within the territories of the Ute, Cheyenne, and Arapaho peoples. Further, we acknowledge that 48 contemporary tribal nations are historically tied to the lands that make up the state of Colorado. We recognize and affirm the ties these nations have to their traditional homelands and the many Indigenous people who thrive in this place, alive and strong. We also acknowledge the painful history of ill treatment and forced removal that has had a profoundly negative impact on Native nations. *Modified by ACB from the CU Boulder Center for Native American and Indigenous Studies and the the CU Office of the President.*

In acknowledging these Peoples and these transhistoric lands and waters, it is important to recognize that our ability to have universities on these lands, and to live and work in these places, is due to colonial policies and the practices of genocide and cultural erasure. In making this acknowledgement, we hope to create a space, if even for a few moments, to disrupt colonizing practices and to continue making visible Native Peoples and places as part of our

individual and collective work toward tribal sovereignty, healing, and social transformation. *This broader statement is grounded in writing and work by MH.*
