# Supplementary material for: “Where do I even start?” Recommendations for faculty diversifying syllabi in ecology, evolution, and the life sciences
Source: Ecol Evol. 2023 Jan 3;13(1):e9719. doi: 10.1002/ece3.9719 (PMC9810791; doi:10.1002/ece3.9719)
Supplement: Supplementary file 4 — File S4 [file ECE3-13-e9719-s003.pdf]

Dear [insert resource creator name],

I am [insert professional and/or personal identity], and I have been working with a group of [insert description of your group] to put together a list of resources that can be used as a starting point for instructors who want to diversify and incorporate anti-colonial concepts in the syllabi of their life and environmental science classes. I am getting in touch because one of the sources that we have reviewed was [insert source that was selected to be included]. We were very impressed by your [insert type of resource] and would like to include it in our list, which will be shared via social media. However, we just wanted to get in touch with you to give you the opportunity to let us know if you would rather not have your [insert type of resource] included. If you would like us to remove your [insert type of resource] from our list of resources to share, please let me know and I will make sure to do that.

I have included a draft of our list of recommended resources below for you to look at if you are interested. Your report is listed in the “classroom resources” tab. We would be very grateful if you have the time to review what we have included about you and your resource, and to let us know if there are any changes that you would like us to make. To make this as easy as possible for you, I have also included a link to a short questionnaire. We would ask you not to share the list widely with others at this time, since it is still in preparation.

You will notice that our list of resources also includes information about your racial/ethnic identity if you have made it publicly available. We have included this information in an attempt to encourage instructors to expose their students to researchers and conservationists of diverse backgrounds. One of the questions in the survey asks you whether you would like this information about yourself included, and if so, how you would like your identities to be listed.

Link to Resource List Draft: [insert link to the Blank_external_spreadsheet]
Link to Survey: [insert link to Example Author Feedback Form]

Thank you very much for your time!

Best,

[insert name]
