# Supplementary material for: “Where do I even start?” Recommendations for faculty diversifying syllabi in ecology, evolution, and the life sciences
Source: Ecol Evol. 2023 Jan 3;13(1):e9719. doi: 10.1002/ece3.9719 (PMC9810791; doi:10.1002/ece3.9719)
Supplement: Supplementary file 5 — File S5 [file ECE3-13-e9719-s007.pdf]

Author Info Survey

Name:

Would you like us to include your resource(s) in our list? (Yes/No)

In the draft spreadsheet that we have shared with you, are there any errors in the information about your publication(s) that you would like us to know about? If so, please explain below.

Would you like us to include your contact information in our list? (Yes/No)

If so, please provide your contact information below.

Would you like your racial and/or ethnic identity shared on our list? (Yes/No)

If so, what is your racial and/or ethnic identity?

Is there anything else about your identity that you would like us to share in our list? If so, please provide that information below.

What pronouns do you use?

Is there anything else you would like us to know or anything that you would like us to consider about your resource?
