# Supplementary material for: “Where do I even start?” Recommendations for faculty diversifying syllabi in ecology, evolution, and the life sciences
Source: Ecol Evol. 2023 Jan 3;13(1):e9719. doi: 10.1002/ece3.9719 (PMC9810791; doi:10.1002/ece3.9719)
Supplement: Supplementary file 6 — File S6 [file ECE3-13-e9719-s001.pdf]

Example Feedback Form

We welcome feedback of any kind on these resources, including concerns about the resources provided and suggestions for additional resources that could be included.

Please Share your feedback here:

If you would like to discuss your thoughts, please enter your contact info here:
