# Supplementary material for: “Where do I even start?” Recommendations for faculty diversifying syllabi in ecology, evolution, and the life sciences
Source: Ecol Evol. 2023 Jan 3;13(1):e9719. doi: 10.1002/ece3.9719 (PMC9810791; doi:10.1002/ece3.9719)
Supplement: Supplementary file 7 — File S7 [file ECE3-13-e9719-s004.pdf]

Topic Lead Checklist

Week 1:

- Send out email to group members to solicit resource suggestions:
- Include with the following information:
    - Topic of weekly meeting
      (general description, anything specific you'd like to see)
    - Link to big sheet of where to add resources
    - Deadline for when to submit resources by (1-week before meeting)
- Send reminder email one day before resources are due

Week 2:

- Send out email to group members with the following information at least 5 days before the meeting:
    - List of 3-5 selected resources (est. 1-2 h of reading/listening)
    - Add pdfs of the selected articles to google drive and include link in email
    - Any information, topics or discussion points that you want folks to think about
  - Examples of discussion questions:
    a. Author self-identity
    b. Highlighted Contributions
    c. Ideas about use
    d. Anything problematic?
    e. Do we recommend this paper?

- Send email reminder with zoom link and information one day before meeting
- Make brief Google Slides presentation (1 slide per resource)

Post-meeting (within one week of meeting):

- Move recommended resources to the shareable document/s sheet & update original sheet as needed
- Send out emails to resource creators:
    - Identity information
    - Feedback form
